# METROID: an automated method for robust quantification of subcellular fluorescence events at low SNR

**DOI:** 10.1186/s12859-020-03661-9

**Published:** 2020-07-24

**Authors:** Marcelo Zoccoler, Pedro X. de Oliveira

**Affiliations:** 1 Department of Biomedical Engineering (DEB), School of Electrical and Computer Engineering, University of Campinas, 400, Albert Einstein Avenue, Campinas, SP 13083-852 Brazil; 2 Center for Biomedical Engineering (CEB), University of Campinas, Campinas, SP Brazil

**Keywords:** Blind source separation, ROI generation, Fluorescence images, Independent component analysis, Voltage sensitive dyes

## Abstract

**Background:**

In cell biology, increasing focus has been directed to fast events at subcellular space with the advent of fluorescent probes. As an example, voltage sensitive dyes (VSD) have been used to measure membrane potentials. Yet, even the most recently developed genetically encoded voltage sensors have demanded exhausting signal averaging through repeated experiments to quantify action potentials (AP). This analysis may be further hampered in subcellular signals defined by small regions of interest (ROI), where signal-to-noise ratio (SNR) may fall substantially. Signal processing techniques like blind source separation (BSS) are designed to separate a multichannel mixture of signals into uncorrelated or independent sources, whose potential to separate ROI signal from noise has been poorly explored. Our aims are to develop a method capable of retrieving subcellular events with minimal a priori information from noisy cell fluorescence images and to provide it as a computational tool to be readily employed by the scientific community.

**Results:**

In this paper, we have developed METROID (Morphological Extraction of Transmembrane potential from Regions Of Interest Device), a new computational tool to filter fluorescence signals from multiple ROIs, whose code and graphical interface are freely available. In this tool, we developed a new ROI definition procedure to automatically generate similar-area ROIs that follow cell shape. In addition, simulations and real data analysis were performed to recover AP and electroporation signals contaminated by noise by means of four types of BSS: Principal Component Analysis (PCA), Independent Component Analysis (ICA), and two versions with discrete wavelet transform (DWT). All these strategies allowed for signal extraction at low SNR (− 10 dB) without apparent signal distortion.

**Conclusions:**

We demonstrate the great capability of our method to filter subcellular signals from noisy fluorescence images in a single trial, avoiding repeated experiments. We provide this novel biomedical application with a graphical user interface at 10.6084/m9.figshare.11344046.v1, and its code and datasets are available in GitHub at https://github.com/zoccoler/metroid.

## Background

In biological and medical sciences, a continuous challenge to quantify fast biological events in space is the reliable signal extraction from images through which a physical quantity can be obtained. To that purpose, a great variety of fluorescent probes has been developed whose response could specifically correlate to a sought variable such as ion concentration, pH, electric field, gene expression, proteins interaction and location. In excitable cells, the major variable of interest is membrane electric potential. For decades, scientists have been using voltage sensitive dyes (VSD) to observe and quantify membrane potential because they allow simultaneous multi-site measurements and are relatively noninvasive when compared to microelectrodes. These fluorescent probes bind to cell membrane and show a shift in emission spectra correlated to the local electric field amplitude across the membrane [1]. Historically though, externally applied fast-VSD have displayed low signals (ΔF/F ~ 0.01–10%/100 mV) [2] and have been rarely used to extract signals from isolated cells, mostly due to low signal-to-noise ratio (SNR) and high susceptibility of isolated cells to phototoxic effects [1, 3]. Even with the recent development of genetically encoded protein voltage sensors with improved responses [4], where toxic effects are absent, signals must be reconstructed by exhaustive signal averaging from repeated experiments with different cell samples or different times [5, 6].

Certain situations make repeated experiments unfeasible with the same sample, like in cell electroporation assays by external electrical fields. Cellular membranes abound with polar and charged molecules, which respond to an increasing electrical field pulse by spatially varying their electrical potential in a cossenoidal fashion, depolarizing towards the cathode side and hyperpolarizing towards the anode side [7, 8]. Once this membrane potential reaches a certain threshold, usually at one or both cell edges, electroporation can be inferred by a rapid and persistent membrane potential transition from threshold values towards zero all over the membrane, which is attributed to a drastic increase in membrane permeability by means of pore formation [9]. This experiment configuration is hardly repeatable for two main reasons: cells either die after successive shocks from cumulative damage or they recover after several minutes, but activate membrane repair mechanisms that make their response to a second membrane disruption different [10]. Therefore, electroporation measurements usually depend on a single trial per sample, which renders high fluorescence signals essential.

Alongside with new fluorescent probes development and modern microscope designs, quantification of membrane potential benefits from improved image and signal processing methods. A common first step in fluorescence imaging measurements is to select regions of interest (ROI) comprehending local events as an attempt to highlight a desired signal. In spite of this, the simple fact that a smaller area is selected accentuates noise power due to a decreased shot noise spatial averaging. If the local event amplitude is not high enough, ROI signals may be overflown by noise and the sample may be either discarded or may demand repeated measurements for later temporal averaging. Instead of this, signals from a single sample could be filtered prior to quantification.

A classic way to remove unwanted frequencies is with digital filters. However, it is usually difficult to determine filter cutoff frequencies adequate to remove noise without distorting cellular signal, because signal frequency components may be unknown. Time-frequency analysis like wavelet analysis could help at identifying relevant frequencies because they are able to display frequency components in a time-dependent manner, facilitating signal frequency determination when signal manifestation in time is known. Indeed, such methods have proven more effective than digital filtering to extract physiological signals [11, 12], but they are still sensitive to the mother wavelet, scale selection and coefficients thresholding algorithm [13].

Another set of techniques that has become very popular to separate physiological signals is Blind Source Separation (BSS). These methods require almost no a priori knowledge of the underlying sources of signal besides some reasonable assumptions like noncorrelation or statistical independence [14]. To accomplish this challenging task, BSS methods rely on statistical signal processing and information theory rather than decomposing signal into frequency components. Therefore, they are better suited to analyze non-stationary signals, which is often the case of cellular signals.

In this work, we have performed an extensive analysis of VSD fluorescence signal extraction, from ROI design to real data application, yielding the Morphological Extraction of Transmembrane potential from Regions of Interest Device (METROID). It includes a consistent and automatic method for ROI generation followed by ROI signal filtering by means of BSS techniques. To test its effectiveness, we have compared four BSS techniques performances by means of computer signal processing simulations and we have implemented these simulation optimal results into simulated imaging data and into experimental data composed of fluorescence images produced by exposing isolated cardiomyocytes loaded with VSD to external electric field stimulation. METROID is made available to a broad audience: for developers, its code is freely available online as jupyter notebooks, and for non-programmers, a version with a graphical interface is supplied.

## Results

### METROID general workflow

**Fig. 1:**
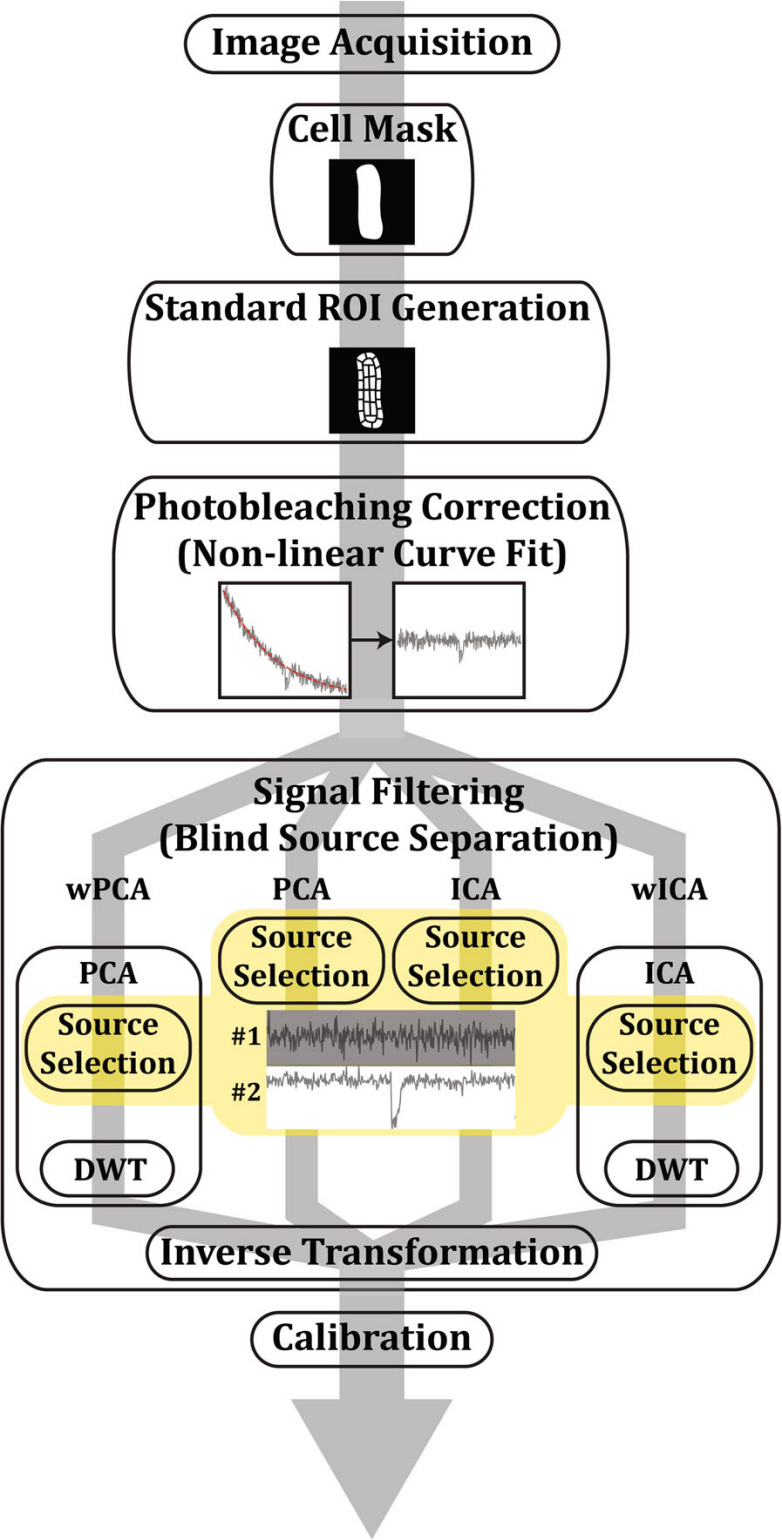
Schematic of the proposed method used by METROID to remove noise from cell fluorescence image. After recording images and creating a cell mask, regions of interest (ROIs) are automatically generated respecting cell shape. Then photobleaching is removed by non-linear curve fit. Finally, one of four blind source separation methods (PCA, ICA, wPCA or wICA) is used to separate signal and noise into different channels. Noise channels are discarded and signal is rebuilt with the remaining source for subsequent data calibration

The method is composed of three main steps: automatic ROI generation, photobleaching correction and signal filtering (Fig. 1). ROI generation is automatically done over a cell mask, which can be also automatically generated or can be manually drawn and edited. The procedure uses a series of classic image processing techniques such as morphological operators [15], image rotation and discrete geometry to divide the mask into quasi-isometric regions. ROIs arise in a standardized fashion, shaped to each cell particular contour, but maintaining a similar area and location, which makes ROIs easier to compare among samples of different shapes. Then, photobleaching removal from each ROI is done by non-linear curve fit. After that, signal filtering is achieved by applying a BSS method using ROI means as multiple observations of a cellular event contaminated by noise. We have compared the performance of Principal Component Analysis (PCA) [16] and Independent Component Analysis (ICA) [17]. Yet, from very early simulations, it was clear that some part of the noise would inevitably remain in the selected source, therefore, as an attempt to further eliminate noise, we have also evaluated a complementary version of these techniques by filtering out higher frequencies of the selected source by means of Discrete Wavelet Transform (DWT) [18]. We have applied DWT onto ICA (wICA) or PCA (wPCA) selected source to eliminate residual noise.

After one of the BSS methods mentioned above was applied, ROI means were rebuilt by applying the inverse transformation using only a selected source containing the signal of interest. This way, noise was removed by discarding noise channels before rebuilding observations (plus eliminating some higher frequency components from the selected source with DWT when wICA or wPCA were used). In the end, a calibration method was done to convert signals from arbitrary units (A.U.) to millivolts.

### Regions of interest results

**Fig. 2:**
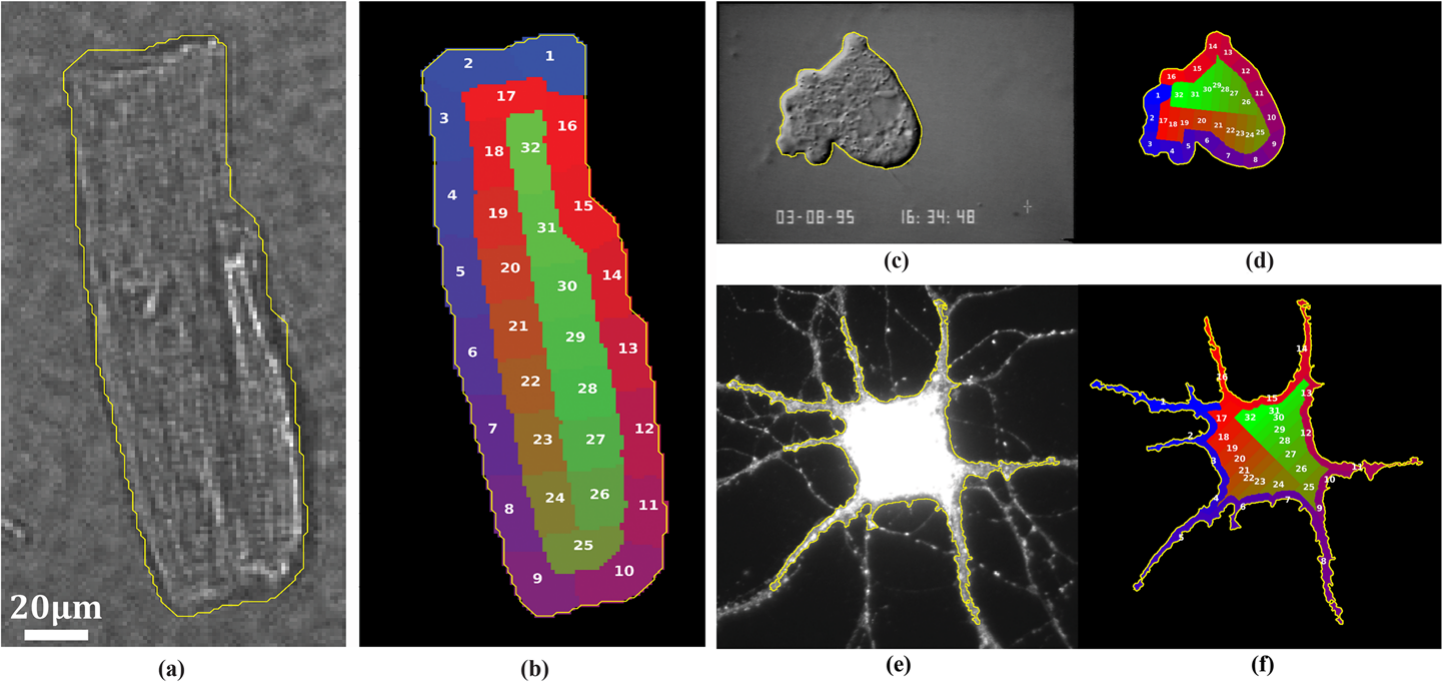
Example of Morphological Equal-area Standardized Segmentation (MESS) application to some cell types and shapes. **a** Isolated rat cardiomyocyte image. **b** Cardiomyocyte standardized ROIs. **c** Ameboid cell (an image of “Shinya Inoue (2011) CIL:11941, Dictyostelium discoideum, amoeboid cell. CIL. Dataset.” (http://cellimagelibrary.org/images/11941), used under CC BY-NC-SA 3.0 (https://creativecommons.org/licenses/by-nc-sa/3.0/legalcode)) overlaid by contour of cell mask. **d** Ameboid cell standardized ROIs. **e** Rat multipolar neuron (“Tonya Anderson, Deanna Benson (2010) CIL:807, Rattus, multipolar neuron. CIL. Dataset.” (http://cellimagelibrary.org/images/807), public domain) overlaid by contour of cell mask. **f** Neuron standardized ROIs

We called our ROI design procedure as Morphological Equal-area Standardized Segmentation (MESS). MESS was designed to deal with ellipsoidal cells, like the cardiomyocyte picture shown in Fig. 2a, but we illustrate its application to two other cell types to test its limits. We show in Fig. 2b the result of dividing the cardiomyocyte shape (yellow contour in Fig. 2a) into 32 ROIs. We show in Fig. 2c and d the application to an ameboid cell DIC image and in Fig. 2e and f the application to a strongly asymmetric neuron cell fluorescence image, where we have applied a different number of inner and outer ROIs. In all cases, MESS fully divides the mask into inner and outer ROIs in a standardized fashion, which is image rotation independent. In the cardiomyocyte and ameboid cells, quasi-isometric division was obtained, but in the neuron, this was not possible because dendrites and axons greatly contribute to the outer mask area.

### Signal processing simulation results

**Fig. 3:**
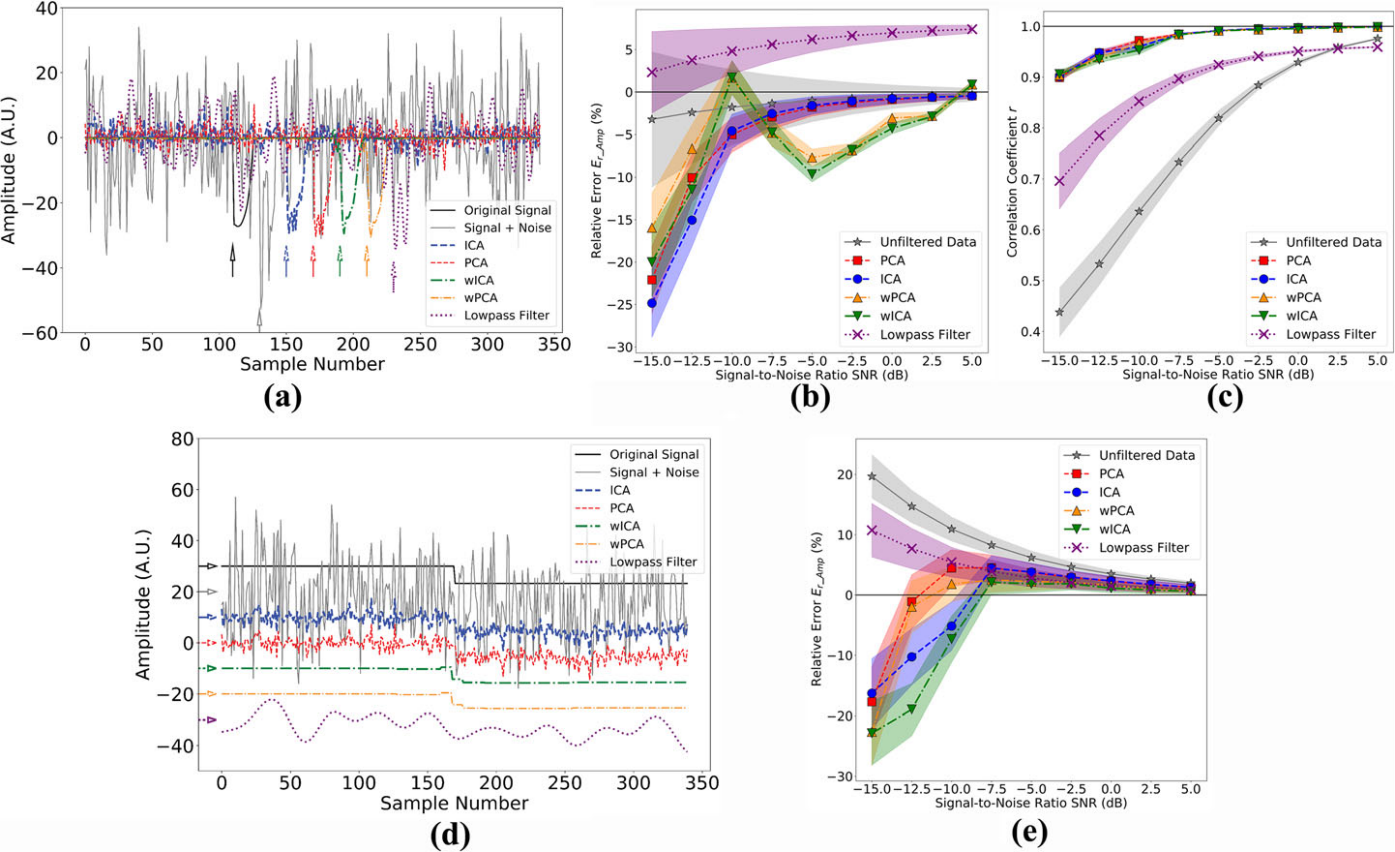
Signal extraction by different filtering methods. **a** Representative simulated AP trace recovered by different BSS methods. Signals are time-shifted for better visualization. Black continuous line is the original noise-free AP signal, gray continuous line is a noisy observation (SNR = − 10 dB), blue dashed line is ICA output, red dashed line is PCA output, green dot-dashed line is wICA output, orange dot-dashed line is wPCA output and purple dotted line is 5th order low-pass Butterworth filter. Matching style arrows indicate AP signal onset time. **b** Relative error for maximal AP amplitude (E_r_Amp_) as function of SNR for AP signal. Gray stars represent E_r_Amp_ means calculated directly from noisy data (*N* = 120 samples for each mean), blue circles represent ICA means, red squares represent PCA means, green inverted triangles represent wICA means, orange triangles represent wPCA means and purple crosses represent 5th order low-pass Butterworth filter. Shaded regions correspond to standard errors of the mean. **c** Correlation coefficient (*r*) means for AP signal calculated in AP active interval (N = 12 samples for each mean). **d** Representative simulated step recovered by different methods. Signals are amplitude-shifted for better visualization. Line color and styles represent the same outputs as in (**a**). Matching style arrows indicate step signal baseline amplitude. **e** E_r_Amp_ for step function

We have filtered manufactured AP or step signals contaminated by noise with each of the BSS methods and with a classical lowpass digital filter, evaluating their performances by error and correlation measures. We show in Fig. 3a one noisy AP observation (SNR = − 10 dB, continuous light gray), the original AP signal without the added noise (continuous black line), and the recovered signals from each method (dashed and dot-dashed color signals). Each signal was time-shifted for better visualization and a matching style arrow indicates each signal starting point. We present the filtering methods relative error for AP maximal amplitude (E_r_Amp_) as a function of SNR in Fig. 3b and Pearson correlation coefficient (*r*) in Fig. 3c. The shaded regions represent standard errors of the mean. Around − 7.5 dB, ICA and PCA E_r_Amp_ means become closer to unfiltered data mean, but with a much smaller variability and a much higher correlation with the original signal (r > 0.9 since -15 dB, Fig. 3c), which can also be inferred from representative traces in Fig. 3a. Figure 3d shows the same situation for the step observation and, in this case, each signal was amplitude-shifted for better visualization and a matching style arrow indicates the shifted baseline. Figure 3e contains E_r_Amp_ for the step as a function of SNR. The modulus of E_r_Amp_ amplitude mean is smaller than unfiltered data mean since -10 dB, and seems to approach zero at smaller SNR for PCA and wPCA than for ICA and wICA. For both signals, we can see that wavelet-based methods better resemble original signals, which is consistent with removal of extraneous noise that remained in the selected source channel. This is counterbalanced by an increase of the modulus of E_r_Amp_ mean values at some SNR in AP simulations. The lowpass filter applied to AP signal also resulted in small E_r_Amp_ means (less than 10%), but signal deformation is clear and even more evident, as expected, for step signal.

Root-mean-squared errors (RMSE) as a function of SNR were also calculated and are presented in Additional file 1. The results for the simulations where, instead of varying SNR, we have varied the number of components (*m*) and the number of observations (*n*) are provided as supplementary materials (Additional files 2 and 3, respectively). Overall, these figures reveal that a lower *m* and a higher *n* tend to give smaller errors and bigger correlations. In particular, *m* = 2 and *m* = 3 give the smallest errors, and *n* > 30 tends to hamper error increase.

Similar simulations with an added exponential function were run at SNR = − 10 dB, mimicking photobleaching presence. The photobleaching compensation performance was evaluated by fitting noisy data with the same exponential function, but based on three different noisy data portions: before signal onset, which we called Single Bind Photobleaching Correction (SBPC), before signal onset plus after signal end, which we called Double Bind Photobleaching Correction (DBPC) and whole data, including signal, which we called Full Data Photobleaching Correction (FDPC). SBPC and DBPC were applied to AP simulations because it is a transitory signal, and SBPC and FDPC were applied to step simulations. Examples of each photobleaching correction are provided in Additional file 4. Results from relative errors of fitting exponential parameters are shown in Additional file 5 and the impact of each photobleaching correction over each BSS method applied afterwards is shown in Additional file 6. In almost every situation, SBPC displayed a worse performance while the other photobleaching correction methods were either similar to results without photobleaching (mainly observed in relative errors) or slightly, but significantly different (mainly observed in RMSE). When either DBPC or FDPC were used, the modulus of E_r_Amp_ mean of every BSS method was always smaller than 8%, while using SBPC could make these means reach values as high as 26%.

### METROID interface

METROID code and experimental data are freely available in jupyter notebook format at https://github.com/zoccoler/metroid and its version with a graphical interface can be downloaded at 10.6084/m9.figshare.11344046.v1. The user must provide the following mandatory inputs:

1. Path to directory containing the fluorescence video or cell mask. Videos should be “.tif” grayscale stack images and masks should be “.tif” binary images (optionally masks can be manually drawn over an image);
2. Video frame rate;
3. Whether signal should be treated as transitory or persistent. In the jupyter notebook code, this is done by the input variable called ‘transitory’, while in the graphical interface, this is done by a drop-down list. This variable is important for two reasons: first, to select the photobleaching correction method to be used (DBPC or FDPC) when bleaching compensation is desired, and second, as a guide to detect and estimate signal active interval, used to drive automatic component selection.

Many optional parameters may be provided, which may improve signal extraction quality (default values or strategy used to obtain them are shown in following parenthesis): number of inner ROIs (16), number of outer ROIs (16), time of signal onset (time of biggest modulus of derivative calculated from whole cell signal mean over time), time of signal ending (time where whole cell signal mean over time falls below estimated noise), number of BSS components (1), BSS method (ICA), wavelet (*Haar*, not used if method is ICA or PCA), and manual or automatic source selection (‘auto’). If source selection is set to manual, all sources are plotted, execution is halted and more than one source may be selected by the user before BSS inverse transformation.

**Fig. 4:**
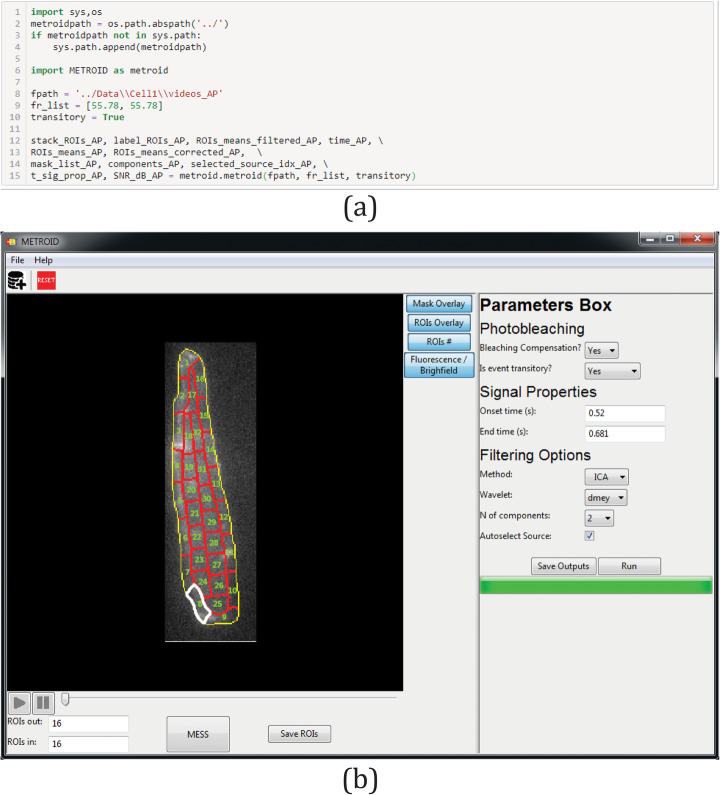
METROID interfaces. **a** Block of code in jupyter notebook format to import and run METROID to process two AP videos. **b** Main window of METROID graphical user interface version after an AP video had been processed

We present the two possible interfaces supplied for METROID in Fig. 4. In Fig. 4a, we show how METROID can be executed on two AP videos with a few lines of Python code. Briefly, METROID path is appended to the interpreter’s search path, then METROID is imported, mandatory input variables are set and finally the main METROID function (also called metroid) is called with the mandatory inputs and preceded by output variables. In Fig. 4b, we show METROID with its graphical interface after a video has been processed. The video can be played in the left panel and each ROI filtered signal over time can be displayed as a graphic in a new window by double-clicking on the respective ROI. Right panel allows the user to provide input variables and all outputs can be saved in an output folder once execution is completed. Some other editing options are: cell mask can be drawn manually, ROIs can be imported from an external file compatible with ImageJ’s ROI Manager tool output and photobleaching correction can be disabled.

### METROID performance on simulated imaging data

**Fig. 5:**
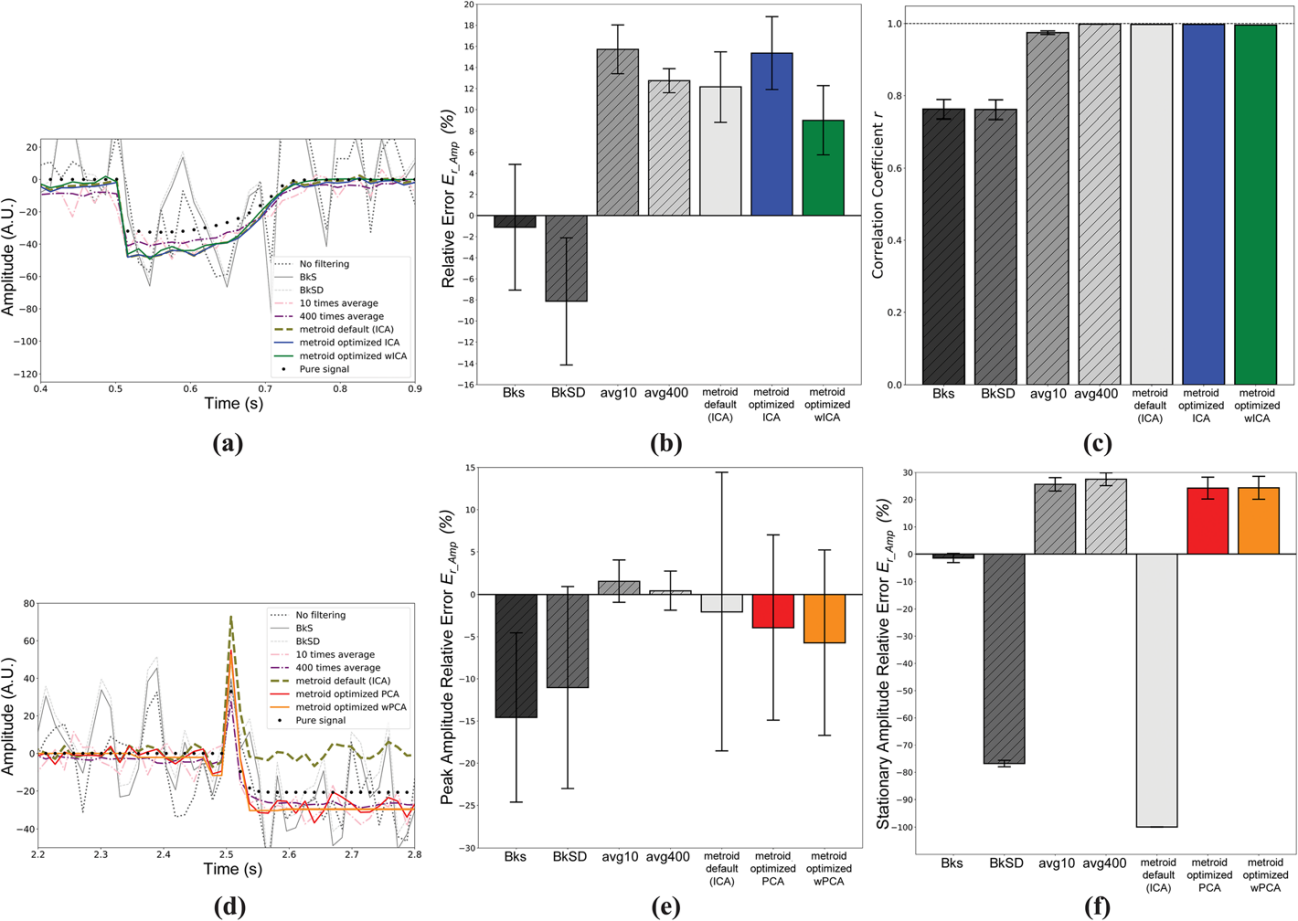
METROID comparison to other methods on simulated imaging data (SNR similar to experimental data). **a** Representative traces in a ROI of AP filtered signal by different methods. Black circles represent true AP signal, dark gray dotted lines are ROI mean over time, gray continuous lines are BkS, light gray dashed lines are BkSD, pink dot-dashed lines are the average of 10 videos (avg10), purple dot-dashed lines are the average of 400 videos (avg400), olive green thick dashed lines are METROID output with default parameters, blue continuous lines are METROID optimized with ICA (onset and end times supplied), and green continuous lines are METROID with wICA (onset and end times supplied). **b** E_r_Amp_ means of each method (*N* = 32). Vertical lines represent standard error of the means. **c***r* means. **d** Representative traces in a ROI closer to the cathode side of electroporation filtered signal by different methods. Same color scheme as in (**a**), except that red continuous lines are METROID optimized with PCA (onset and end times supplied, *m* = 3), and orange continuous lines are METROID optimized with wPCA (onset and end times supplied, *m* = 3). **e** Peak E_r_Amp_ means. **f** Stationary E_r_Amp_ means

We have generated simulated fluorescence videos, where we have complete knowledge about the underlying signal, to validate our method. The videos consist of the sum of a basal fluorescence profile image, a membrane potential signal (AP or electroporation) modulated for a desired SNR, a photobleaching function and simulated shot noise. We show in Fig. 5 the performance comparison of METROID with several other methods on a simulated imaging data with the same SNR distribution per ROI and the same number of ROIs (32) as the experimental data presented in the next section. The number of ROIs was chosen as 32 because, from signal processing simulations, *n* > 30 has proven to be enough to hamper error increase. The other methods used for comparison were either derived from flat-field correction and detrending methods, like blank subtraction (BkS) and blank subtraction with detrending (BkSD, analog to [19]), or based on classical repeated signal averaging, like the time average of 10 videos (avg10) and of 400 videos (avg400, performed experimentally in [6]). METROID was run with its default parameters and with two settings of optimized parameters (method selection and signal onset and end times provided). In Fig. 5a, we show representative traces of one of the ROIs average over time (ROI #1, closer to the anode side). Pure AP signal is displayed as black circles and unfiltered trace is shown as dark gray dotted lines. BkS is shown as continuous gray lines, BkSD is shown as dashed light gray lines, avg10 is shown as pink dot-dashed lines, avg400 is shown as purple dot-dashed lines, METROID default is shown as thick olive green dashed lines, METROID with ICA is shown as blue continuous lines and METROID with wICA is shown as green continuous lines. In Fig. 5b, we show E_r_Amp_ means of each method and, in Fig. 5c, we show *r* means of each method with vertical bars representing standard errors of the means. Non-parametric Kruskal-Wallis test indicated a significant difference among E_r_Amp_ means, with Dunn’s post-test only indicating adjusted significant *p*-values (*P* < 0.05) when comparing BkSD to avg10, and BkSD to avg400.

In Fig. 5d, we show representative traces of the same ROI, but for simulated electroporation video. The methods, line styles and color scheme are the same as Fig. 5a, except that METROID optimized methods were changed from ICA to PCA, shown in red, and from wICA to wPCA, shown in orange, both with onset time provided and *m* = 3 to allow 2 sources to be selected and 1 noise to be discarded. These choices were done based on better performances shown by each method in previous signal processing simulations. We show, in Fig. 5e, the peak E_r_Amp_, which describes the amount of error from the true peak in each ROI, and we show, in Fig. 5f, the stationary E_r_Amp_, which describes the amount of error from the true median value after signal onset (same definition as in signal processing simulations, i.e., relative error from step amplitude). Kruskal-Wallis test on peak E_r_Amp_ means failed to indicate statistical difference. One-way analysis of variance indicated statistical difference for stationary E_r_Amp_ means, with Tukey’s post-test showing significant adjusted *p*-values when comparing BkS with all others, BkSD with all others and METROID default with all others.

RMSE means of each method can be visualized in Additional file 7. We have also analyzed a similar artificial video without photobleaching (Additional file 8), an artificial video with photobleaching, but with poorer SNR per ROI (SNR = − 10 dB in all 32 ROIs, Additional file 9) and one last artificial video without photobleaching with this same low SNR = − 10 dB (Additional file 10).

### METROID performance on VSD imaging real data

**Fig. 6:**
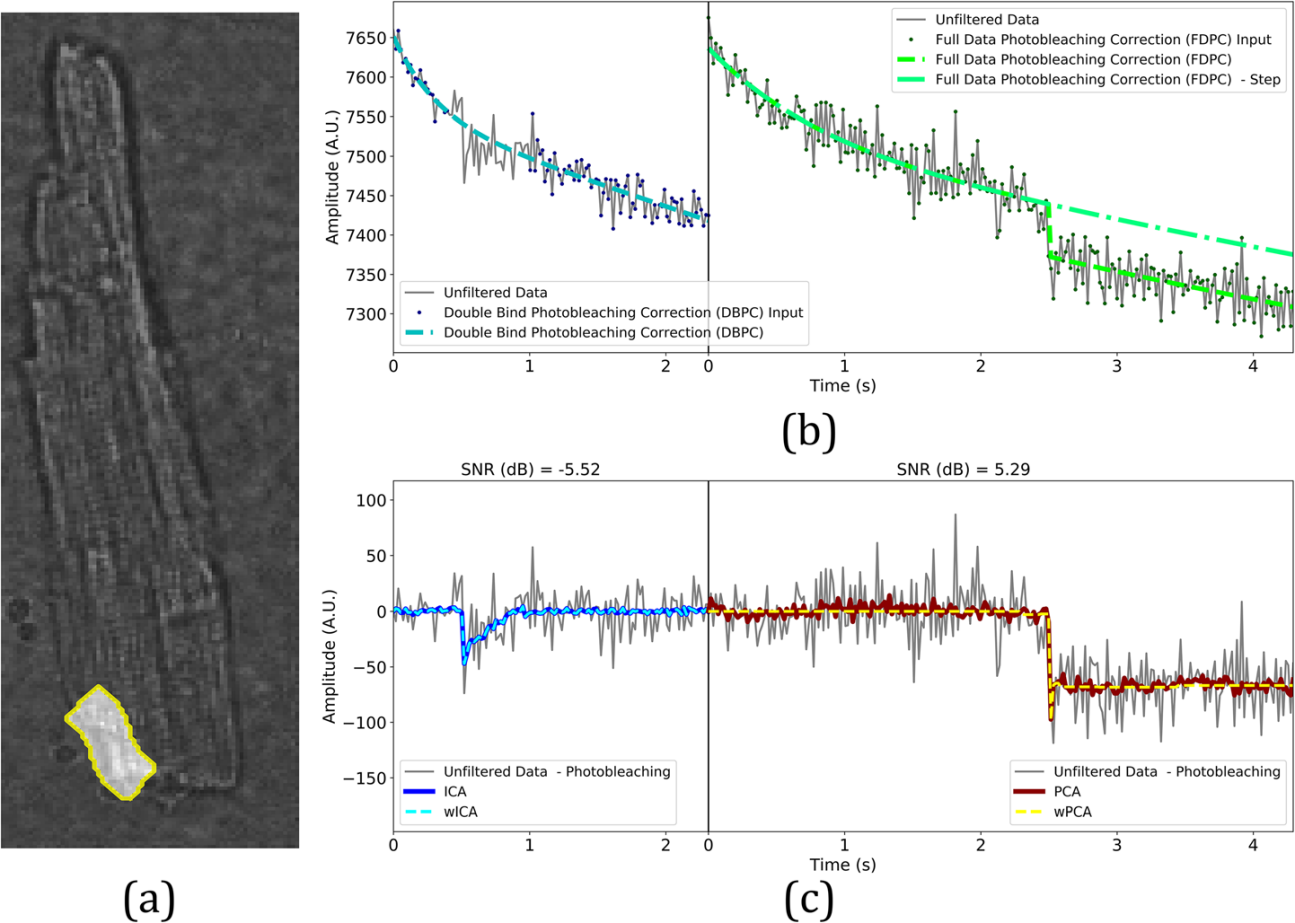
Experimental VSD imaging real data application. **a** Bright-field isolated cardiomyocyte image overlaid by one ROI. **b** ROI means photobleaching correction for AP experiment (left graphic) and electroporation experiment (right graphic). Gray continuous lines are ROI means over time, dark blue or dark green dots are photobleaching correction input data, dashed light blue line (left graphic) is Double Bind Photobleaching Correction (DBPC), dashed light green line (right graphic) is Full Data Photobleaching Correction (FDPC), and dot-dashed green line is FDPC without step. **c** Blind source separation application after photobleaching correction for AP (left graphic) and electroporation (right graphic) experiments. Gray continuous line are ROI means fixed for photobleaching, thick blue continuous line (left graphic) is ICA output, light blue dashed line is wICA output, thick dark red continuous line is PCA output (right graphic), and orange dashed line is wPCA output. Estimated signal-to-noise ratios (SNR) are shown above each graphic

We have applied METROID onto real data generated from experiments with isolated cardiomyocytes loaded with a potentiometric dye under external electric filed stimulation. METROID took 1.67 s ± 0.07 s (mean ± SD) to complete processing a video of 130 frames after parameters definition. Figure 6 is an example of photobleaching correction followed by BSS application in an isolated cardiomyocyte, but it is also a composite version of the window displayed once the user double-clicks a ROI. In Fig. 6a, cell bright-field image is overlaid by the selected ROI. In Fig. 6b and c, the graphics to the left were generated from experimental AP video and the graphics to the right were generated from experimental electroporation video. In Fig. 6b, we show ROI means over time as light gray continuous curves, data input for DBPC as dark blue dots and the DBPC output as a light blue dashed curve. In Fig. 6b right graphic, we also show data input for photobleaching correction as dark green dots (in this case corresponding to the entire data) and FDPC output as a light green dashed curve. The curve that actually represents photobleaching does not have a step, so we show FDPC without the step as a dot-dashed green curve and this is the curve that is subtracted from noisy data to fix photobleaching.

In Fig. 6c left graphic, we show noisy data corrected by DBPC as gray continuous curve, ICA output as thick continuous blue curve, wICA as light blue dashed curve and the estimated SNR above the graphic. The estimated SNR for all ROIs stood between − 13.31 dB and 3.29 dB, with an average of − 1.54 dB. ICA reduced noise power by an average of 97.24%, whereas wICA reduced it by an average of 94.14%.

In Fig. 6c right graphic, we show noisy data corrected by FDPC as gray continuous curve, PCA as thick dark red continuous curve, wPCA as yellow dashed curve and estimated SNR above the graph. Overall, estimated SNR remained between − 4.33 dB and 8.47 dB with an average of 4.24 dB. PCA reduced noise power by an average of 96.23%, whereas wPCA reduced it by an average of 99.93%. The same figure portraying the other ROIs results can be viewed as a movie (see Additional file 11).

**Fig. 7:**
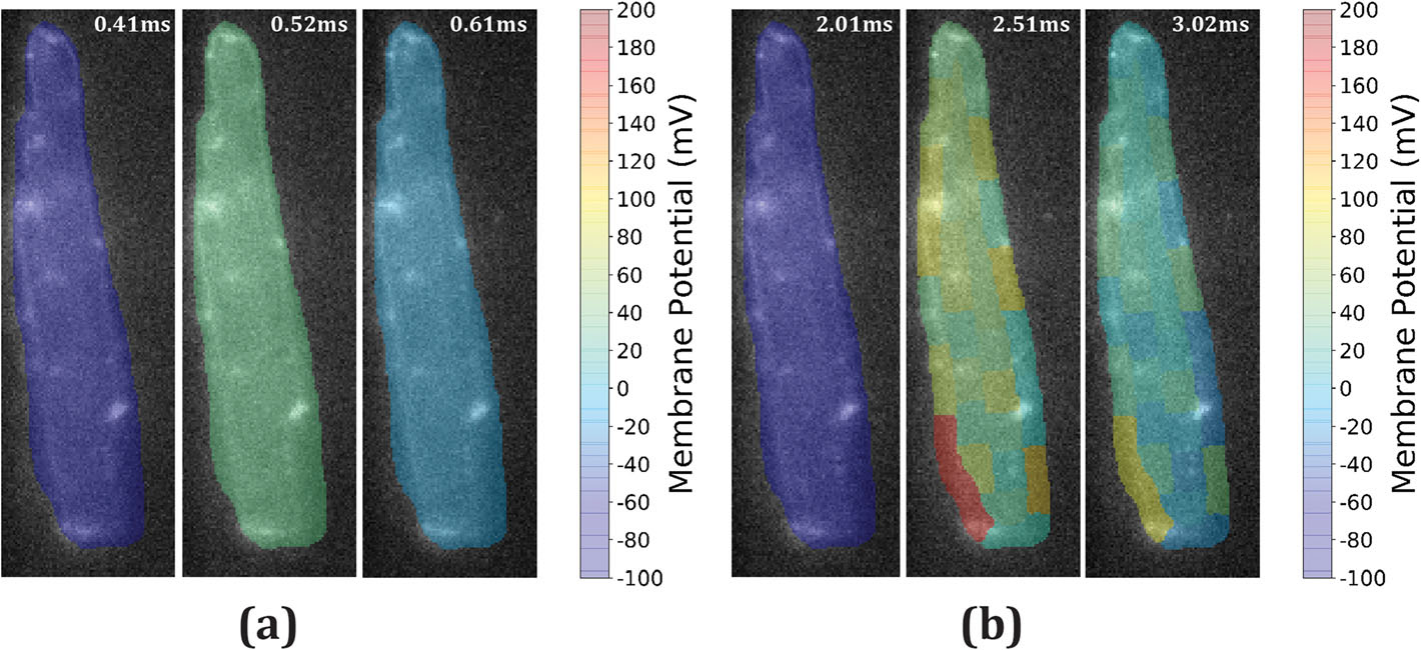
Cell membrane potential variation. **a** Cell standardized ROIs filled with colors representing membrane potentials before AP triggering (left), at AP peak (middle) and at AP plateau (right). **b** Cell ROIs with calibrated membrane potentials before electroporating pulse (left), at signal peak (middle) and after pulse (right)

In Fig. 7a, we show all ROIs generated by ROI standardization filled with a color scale corresponding to membrane potential amplitude. Three time instants were chosen to show membrane potential: before AP (Fig. 7a left image), at AP peak (Fig. 7a middle image), and in plateau phase Fig. 7a right image). AP signal was filtered by ICA here and AP peak was 40 mV by calibration design. We have measured duration at 25 and 75% repolarization (APD_25_ and APD_75_), obtaining APD_25_ = 35.8 ms and APD_75_ = 251 ms. Then, in Fig. 7b, we also show membrane potential for electroporation video filtered by wPCA before strong stimulus pulse (Fig. 7b left image), at electroporation peak instant (Fig. 7b middle image), and after electroporation (Fig. 7b right image). The highest membrane potential levels (V_max_) obtained were 166.18 mV and 168.28 mV in the ROIs that achieved red color levels.

## Discussion

### Regions of interest design

MESS has proven to be an easy and robust method to generate ROIs systematically, regardless of cell shape and orientation. Cytoplasm heterogeneity is represented in a mosaic-like division, but split into inner and outer ROIs. This feature has allowed membrane potential analysis around cell contour, which is often sought in experiments with externally applied electric fields [8, 20, 21], without wasting information coming from inner regions.

Although not designed for strongly irregular shapes like neurons, MESS could still be applied if the sole purpose is to generate subcellular samples, as demonstrated in Fig. 2. Since we have the presence of dendrites and axons that greatly contribute to cell external perimeter, the number of morphological erosions necessary to achieve half area is relatively small, which compromised outer ROIs area equality, because ROIs that represent dendrites or axons (ROIs 1, 5, 8, 11, 14 and 16) retained a higher than average area while ROIs in between were left with lower than average area.

MESS has some limitations, starting with the fact that the method is sensible to motion artifacts since ROIs are static. Besides that, user has no specific control about ROIs springing place. If the user wants to change a ROI location, a possible approach is to change the number of outer ROIs (*n_ROIs_out*) and/or the number of inner ROIs (*n_ROIs_in*), which changes their area and location simultaneously. However, MESS is not designed to detect and delineate sparse subcellular events in space, like calcium sparks for instance. For that goal, we suggest users to use softwares that generate ROIs based on image content [22, 23]. Finally, there are limits to the number of ROIs: *n_ROIs_out* ≥ 2, *n_ROIs_in* ≥ 4 and *n_ROIs_in* must be even. The maximum number of ROIs will depend on image resolution.

### Signal processing simulations

Our simulations have shown that we were able to retrieve a single source containing cellular information blended in a set of random shot noises. From our results, it was clear that any BSS method could outperform digital filtering at SNR ≥ − 10 dB if we consider E_r_Amp_ and *r* (Fig. 3b, c and e). Digital filters also demand anticipated signal frequency components knowledge, while with BSS methods, the single preceding information that we have anticipated was signal onset time in order to allow automatic source selection. In cases where signal onset information is absent, source selection can be done by visual inspection as already performed in other studies [24].

To compare among BSS methods, we should first consider in what conditions each of them would stand out. Briefly, PCA converts the original observations into an orthogonal linear basis. If the underlying sources are uncorrelated, this usually highlights signal from noise, especially if they have normal distributions and distinct variances. On the other hand, ICA excels at identifying independent sources with nongaussian distributions and it is expected to work properly even with one gaussian distribution source in the mixture [17]. Now, in our simulations, the original signals chosen had a skewed (AP) or bimodal (step) distribution, from which ICA could benefit, but since simulated shot noises approach normal distributions, due to a relatively high λ value from Poisson distribution, it was not clear if ICA would outperform PCA. This may explain, as depicted by Fig. 3, why it is difficult to elicit ICA or PCA as an overall better candidate. Their results are practically the same for *r*, and this also happens for E_r_Amp_ in AP simulations, but in step signal simulations (Fig. 3e), PCA seems better than ICA for SNR ≤ − 12.5 dB (remark that this cannot be stated at -10 dB because their absolute values are very close).

Wavelet-composed methods seemed promising since they would be able to adaptively eliminate noise higher frequencies without affecting signal components. Indeed, from Fig. 3, we can see that restored signals are smoother than those recovered without wavelets. Besides that, correlation seems unaffected when compared to PCA or ICA alone. Nonetheless, there is a striking different behavior when we compare E_r_Amp_ for AP simulations against step simulations: for step simulations, wICA and wPCA errors are close to ICA and PCA, respectively, but, for AP simulations, their performance is worse and wildly variable. It seems likely that our wavelet thresholding method affected some frequencies present in signal range as signal power was increased. Changing some wavelet parameters like the mother wavelet and the thresholding parameter (*K*) could diminish those errors, but this is beyond the scope of this study.

In our photobleaching simulations, we have shown that, as expected, the more information we provide as input, the better success we get from curve fitting: FDPC performs better than DBPC, which performs better than SBPC (Additional file 4). Other solutions could be using low-rejection filters or letting photobleaching participate in BSS as another source. Unfortunately, both of these methods fail: low-rejection filters are unable to completely eliminate trend and also end up distorting the source of interest whereas letting exponential into BSS left part of it blended into the source containing the original source (data not shown).

### Simulated imaging data

METROID E_r_Amp_ on AP artificial imaging data was not different from any other method, which is already an advantage since it is the only option without the need of extra videos. BkS displayed the lowest E_r_Amp_ mean and requires just one extra video sample without stimuli, but it is evident from the representative traces (Fig. 5a) and from *r* results (Fig. 5c) that it fails at recovering the AP signal waveform. METROID also displayed lower RMSE than other methods, except avg400. This scenario persists even in an artificial video with low SNR (Additional file 9), but in this case METROID need the onset and end time information. Intriguingly, the difference between avg10 and avg400 is smaller than expected from signal averaging. This was due to residues from photobleaching compensation since when we analyzed the videos without photobleaching (Additional files 8 and 10), the difference in their E_r_Amp_ means escalates substantially.

For electroporation artificial videos, the signal can be decomposed into two sources: one containing the peak value due to membrane charging before pore formation and the second containing the stationary value corresponding to the situation where rest membrane potential is lost and membrane potential tends towards zero. Both component amplitudes vary spatially, but the peak component has an extra variation due to different membrane charging around the cell, displaying a different direction with respect to the baseline fluorescence. That said, the peak E_r_Amp_ means were not statistically different (Fig. 5e), but once we introduce a uniform poor SNR of -10 dB situation (Additional file 9), we obtain statistical difference among means. This is because a very weak punctual peak failed to be disentangled from noise into a separate component. We have seen that this separation only starts to take places when ROIs SNR > − 3 dB, therefore peak detection should be possible when ROI SNR is within this range. Stationary E_r_Amp_ mean is also statistically smaller for BkS, so its median value should be more reliable to get the stationary value, but we can see from Fig. 5d and Additional file 7 that it still contains a lot of noise and does not resemble the true signal. BkSD displayed in general higher errors than BkS probably because photobleaching was already well compensated in BkS (negligible drift between samples). Lastly, METROID default performs the worst for stationary E_r_Amp_ because it selects only one signal source and, in this case, it took the peak component, leaving the stationary component out and justifying the error of about 100%. However, results from the default situation often provide the information needed to optimize METROID parameters, like signal onset timing and expected number of sources, making possible to reach its optimized results in a subsequent run.

### Experimental imaging data

In a single trial, METROID was capable of extracting and characterizing AP and electroporation signals from small ROIs whose signals would be otherwise impossible to analyze due to elevated noise power. In addition, the process can be fully automated if default parameters are used because ROIs are systematically generated and the component containing the signal is automatically selected based on cell global signal. Therefore, the user can process a video by just providing the video itself, plus the overall ‘transitory’ parameter if photobleaching correction is needed. This is a good strategy if user knows little about the data, but some rerunning with edited parameters may be required for better results, especially if signal onset time is wrong.

Photobleaching correction was very effective, which can be verified by the flat baseline outside signal active interval, and noise was extensively removed (Fig. 6c). Fitting photobleaching by a combination of exponential and linear functions has shown to be a better compromise between goodness of fit and convergence. To check whether filtered signals are in agreement with reports from the literature, we have compared some of their characteristics. AP was longer than reported in the literature for the same cell type (APD_75_ = 46 ± 4.1 ms [25]). This is probably due to phototoxic effects of potentiometric dyes like RH-237 [26, 27], despite our efforts to minimally expose cells to excitation light. In addition, we could not directly assess AP amplitude during the experiments, thus AP amplitudes are relied on literature of same cell type.

In Fig. 7b, we can see two ROIs with strikingly higher membrane potential variation. They are located close to a cell edge facing an electrode since electric field was applied vertically. As such, they could represent electroporated regions with electroporation threshold being 248.28 mV. Other studies have found higher threshold values (300-400 mV [28], 480-650 mV [8]), but a review study shows that thresholds can vary from 200 mV to 1 V [9]. These variations are due to several factors like the technique used (patch-clamp in [28]), sensitivity of the image system, which includes our relatively low frame rates, cell type (rat cardiomyocytes in our work, frog cardiomyocytes in [28] and CHO cells in [8]) or pulse duration (5 ms in our work, 0.2 ms and 1.5 ms in [8]). Despite that, our calculated electroporation threshold levels are well above the maximal AP amplitude and elicited a non-transitory response during the few seconds of our experiments duration, which is an indication of electroporation in cells [29].

Besides displaying a peak value, we were unable to separate it into a different component, which means that either our method failed to identify it as a different component or the peak value was lost during the experiment due to a limited precision of stimulus and camera synchronization (about 10 ms variation). Based on the simulated imaging data, the second option is more likely because at that estimated SNR (mean of 4.24 dB), METROID succeeded at separating the peak from the step in the artificial video with similar SNR.

METROID excels in situations where signals are expected to occur ubiquitously in a cell, as the case of membrane potentials elicited by external electric fields. In cases where cellular signals to be detected are scattered with different waveforms and onset timings, BSS methods are still able to separate them, as reported with calcium fluorescence images and VSD images from neuron networks [23, 30]. In fact, these works use spatial-ICA (sICA) to separate signals in time and space as a way to identify neurons individually. In principle, METROID could be applied to a sparse events scenario if the number of components is increased, similar to the case of the electroporation signal, notwithstanding it was not designed nor tested for sparse signal detection. For such goals, there are several other works whose designs are more specialized for detecting spikes in calcium [23, 31–33] and VSD [19, 30] fluorescence images.

We have found a fairly similar work that used non-negative matrix factorization (another BSS method) to eliminate background noise arising from neuropil using ROIs around cell [34]. The authors generated 4 ROIs around a central ROI comprising a somatic cell of interest and were able to eliminate out of focus fluorescence. Although aiming a similar goal, which is noise removal, their method and METROID are not alike. Their criteria for the component selection was the best match with the signal from the central ROI, while METROID has no such reference and its criteria is based solely on either identifying the component with highest power during signal active interval or visual inspection. Other differences involve the number of ROIs, which can be varied here, and the type of image being analyzed. In essence, the methods described here provide new insights on signal and image processing techniques used to face challenges in fluorescence signal extraction.

## Conclusions

This work presents a new method to assess subcellular membrane potential signals by taking advantage of transforming multiple ROIs inside a cell fluorescence image into distinct signal observations and using them as different channels for BSS algorithms. We have shown from our simulated data and experiments with real data that our method outperformed classic filtering at removing noise from multiple ROIs and that it was capable of recovering, in a single trial, subcellular biological signals that would otherwise need repeated experiments. Therefore, METROID introduces a new practical method to segment a cell mask automatically into ROIs as an effective way to extract cell signals from low SNR fluorescence images.

## Methods

### Regions of interest design

**Fig. 8:**
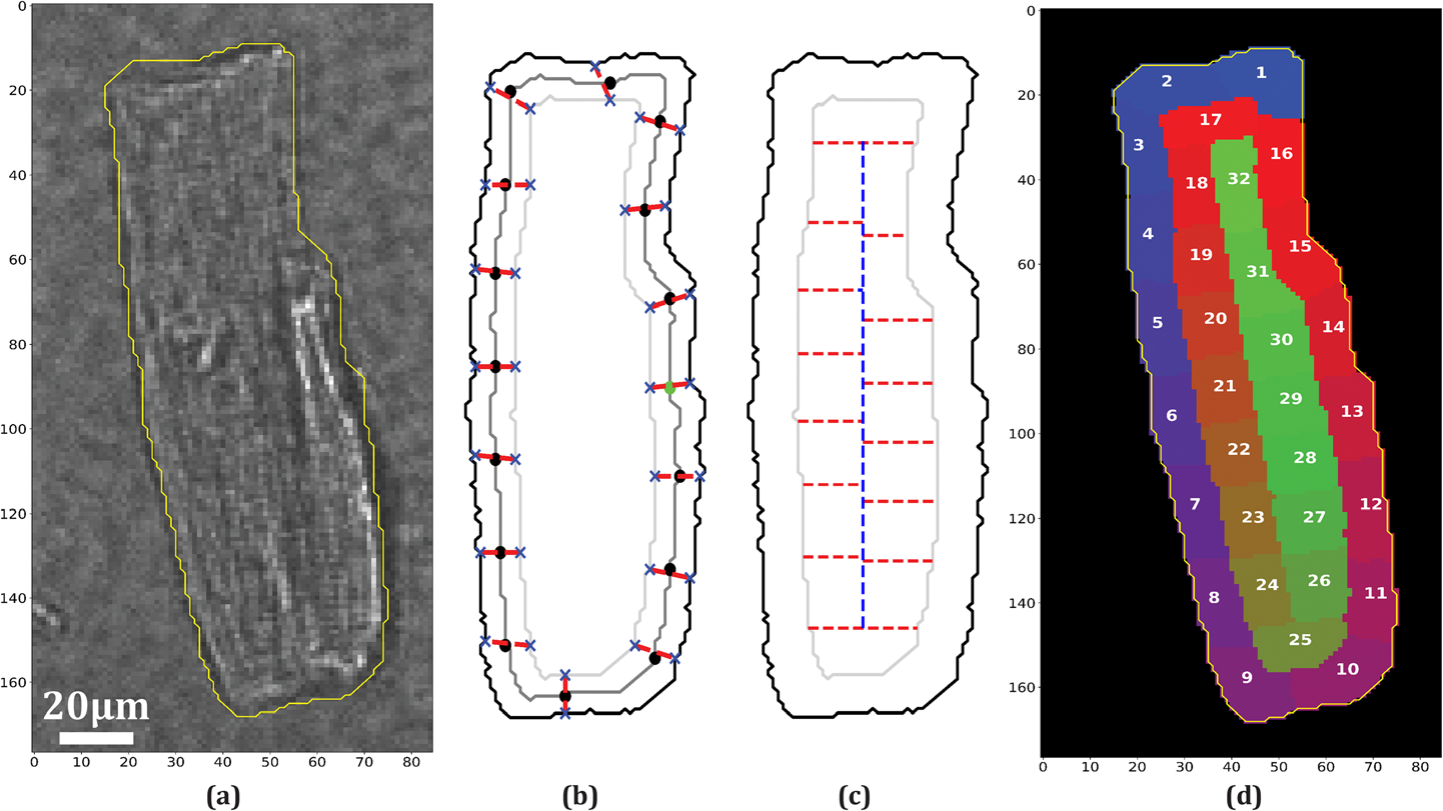
ROI standardization sequence. **a** Bright field rat cardiomyocyte image overlaid by contour representing cell mask. **b** Outer region division: aligned cell outer contour (black contour), mid contour (gray contour) and inner contour (light gray contour). Dashed red lines indicate ROI separation, black dots indicate a segment beginning and blue crosses indicate closest outer and inner contour points. Green dot is the first segment position. **c** Inner region division: aligned outer contour (black contour) and inner contour (light gray contour). Dashed red and blue lines indicate ROI separation. **d** Standardized ROIs represented by different numbers and colors with original cell mask represented by yellow contour

We developed a new method to standardize ROIs called MESS. It has a built-in function to automatically generate a cell binary mask based on the fluorescence image sequence, but it also allows mask manual design or editing. From the binary mask, MESS automatically performs segmentation into ROIs that have approximately the same area and that spring at similar locations, regardless of cell shape and orientation. Figure 8 illustrates the method sequence applied to a cardiomyocyte image. First, it aligns the mask to vertical orientation through image rotation. Then, it initially splits the mask into two regions: an outer region (Fig. 8b, region between black contour and light gray contour) and an inner region (Fig. 8b, region inside light gray contour). This is done by performing sequential morphological erosions from cell mask until the inner area equals approximately (*n_ROIs_in*) / (*n_ROIs_out + n_ROIs_in*) of the total area. From this point forward, each region undergoes a different segmentation method. For the outer region, three contours are defined: the outer contour (the same as the aligned cell contour, Fig. 8b black contour), a mid contour generated by performing half the previous erosions (Fig. 8b gray contour), and an inner contour (the same as inner region contour, Fig. 8b light gray contour). The mid contour is divided into segments of approximately equal lengths (Fig. 8b black dots), and a starting point is selected (Fig. 8b light green dot). This point, regarding the y-axis, is always in the middle of the mask and, regarding the x-axis, is always to the right. From this starting point, coordinates belonging to the outer and inner contours (Fig. 8b blue crosses) closest to this starting point are selected as the beginning of the first ROI and are connected by a line (Fig. 8b red dashed line). This procedure is repeated counter-clockwise at the beginning of each subsequent segment in mid contour. Thus, each outer ROI is bounded by straight lines, by the outer contour, and by the inner contour.

For the inner region, ROI definition goes from top to bottom limited by ROI area, as shown in Fig. 8c by dashed red lines. The only remark in this division is that, except for the top and bottom ROIs, the central area is halved before ROI definition (dashed blue line). The top and bottom inner ROIs are explicitly defined before the others as a design choice to reserve these edge regions because higher signal amplitudes are expected towards cell poles in electroporation experiments. Outer ROIs numbers are shifted by the necessary amount to always leave the first ROI on top of the aligned image. In the end, all ROIs are rotated back to the original cell mask orientation. In Fig. 8d, we show in different colors and numbers the final ROIs. Besides isolated cardiomyocytes, we have applied MESS onto an ameboid cell image and a neuron image to test its operation.

### Signal processing simulations

**Fig. 9:**
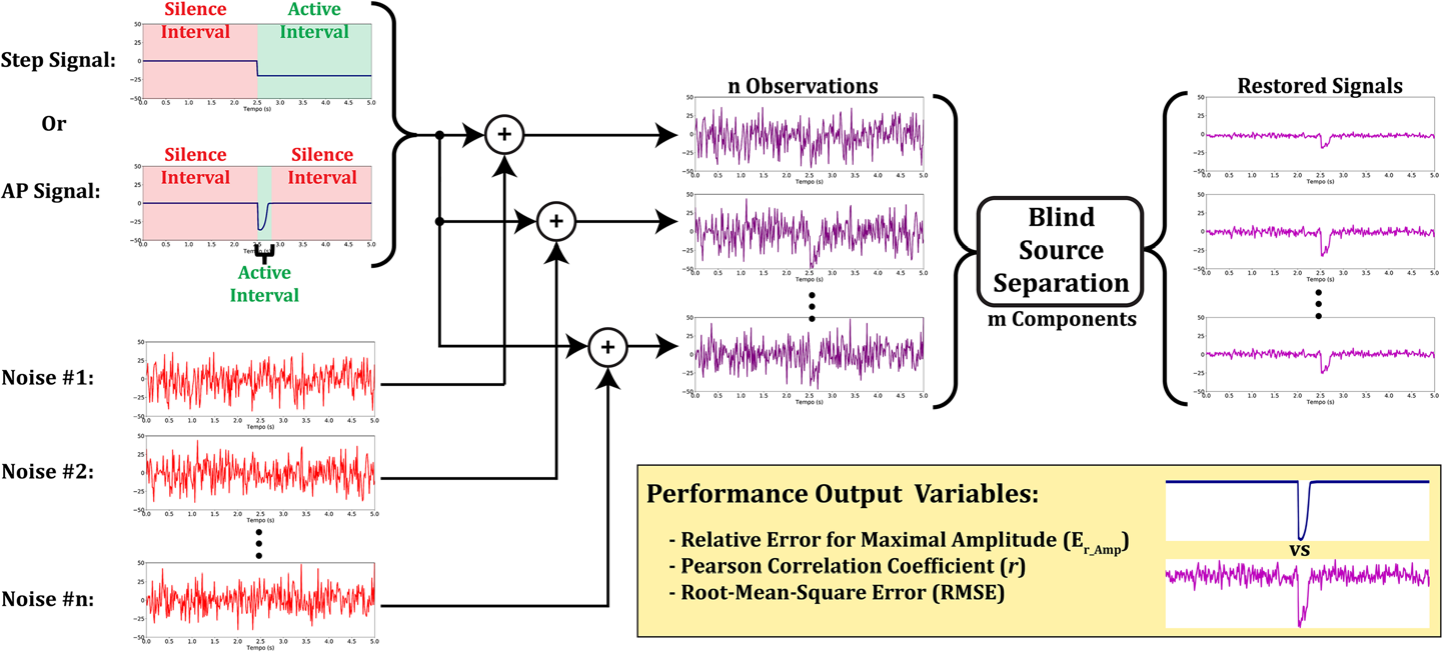
Signal filtering simulations schematic. Each independent noise (red curves) was added to either a step or an AP-like signal (blue curves) to produce *n* observations, simulating a ROI mean over time. These observations served as inputs to one of the four BSS methods, which decomposed them into *m* components. Signals were filtered by applying the inverse transformation with *m* < *n*. Three performance output variables (inset box) were chosen to evaluate each filtering method success: relative error to maximal amplitude (E_r_Amp_), Pearson correlation coefficient (*r*) and root mean square error (RMSE)

We have performed simulations to test filtering methods performance to extract two types of signals associated to transmembrane potentials from noisy observations. The first signal was generated as the output from an online model of ventricular action potential (AP) [35]. The parameters chosen to generate this signal were: “Mahajan et al. (2008) [36] rabbit ventricular cell model”, 0.5 Hz pacing frequency, 1 min maximum pacing time and “0μM compound concentration”. The signal was subsampled to match the frame rate of the real data videos and inverted to resemble the kind of fluorescence variation captured. The second signal is a step signal intended to mimic the situation where a cell membrane was electroporated in response to a strong electric stimulus [29]. Both signals had a total duration of 5 s and were time-shifted to start at 2.5 s, which means that they had a time interval where they were active (active interval: 2.5 to 2.8 s for AP and 2.5 to 5 s for step) and another where they were silent (silence interval: 0 to 2.5 s and 2.8 to 5 s for AP, and 0 to 2.5 s for step). These intervals were used as reference to calculate signal and noise powers afterwards. In Fig. 9 we show a schematic of the simulation steps.

Each observation was the result of the addition of the original source signal (AP or step, blue curves in Fig. 9) to a different independent noise (output from a random Poisson distribution, λ = 225, red curves in Fig. 9) to simulate shot noise. We have subtracted λ from each noise to achieve an approximately zero mean noise. Then, the *n* number of observations served as input to one of the BSS methods which decomposed the observations into *m* components, where *m* ≤ *n*. Whenever *m* is smaller than *n*, this means we are filtering the observations by eliminating some information. Then we have rebuilt the observations with the remaining components by applying the inverse transformation.

We have run three rounds of simulations to analyze the influence of a different parameter in each round. In the first round of simulations, we have varied *m* from 1 to 30 (from 1 to 10 in unity steps and from 10 to 30 in steps of 5) while keeping *n* = 30 and SNR = − 10 dB. In the second round, we have varied *n* from 2 to 60 (from 2 to 10 in steps of 2 and from 10 to 60 in steps of 10) with *m* = 1 and SNR = − 10 dB. In the third round, we have varied SNR from -15 dB to 5 dB in steps of 2.5 dB by varying signal power while keeping noise power constant, *n* = 30 and *m* = 1. In this round of simulations, we have also compared results to a 5th order low-pass Butterworth filter (f_cutoff_ = 10 Hz for AP signal and f_cutoff_ = 2 Hz for step signal). The classical power definition was used for SNR calculation:

1 $$ SNR=\frac{\frac{1}{n_{samples}}{\sum}_i^{n_{samples}}{\left|{x}_i\right|}^2}{{\sigma_{noise}}^2} $$

where *x*_*i*_ is signal value at index *i*, *n*_*samples*_ is the total number of samples in signal duration and *σ*_*noise*_ is noise standard deviation.

To evaluate each filtering method success, we have used three different performance outputs: relative error E_r_Amp_, used to measure accuracy at recovering signal amplitude; Pearson correlation coefficient *r*, used to measure waveform accuracy (just for AP signal during active interval); and RMSE, used to measure precision at noise removal. We have used the following equation for E_r_Amp_:

2 $$ {E}_{r\_ Amp}=\frac{\left({Amp}_{measured}-{Amp}_{true}\right)}{Amp_{true}} $$

where *Amp*_*measured*_ is signal amplitude measured after filtering and *Amp*_*true*_ is the true signal amplitude. This definition is useful because it allows to identify immediately whether signal was overestimated (positive E_r_Amp_) or underestimated (negative E_r_Amp_).

Whenever *m* = 1, component selection was automatically done taking advantage of knowing the event triggering time, which means that all extra components were considered noise and deleted. When using wavelets to filter the selected source, a modified version of fixed hard-threshold *K* [13, 37] was calculated by the following equation:

3 $$ K=\sqrt{2\ln N}\sigma $$

where *N* and *σ* are the number of samples and the median absolute deviation, respectively, in the highest frequency wavelet coefficient. We have used Discrete Meyer (*dmey*) wavelet for AP signal and *Haar* wavelet for step signal because they better correlate to the respective original signals. Each filtered signal had its baseline centered towards zero by subtracting median value obtained from silence intervals.

#### Signal processing simulations with photobleaching

We have also evaluated whether previous photobleaching removal could bias the filtering methods. Thus, we have rerun the third round of simulations, but now observations were a bit different. We have added signal (AP or step), noise, and an extra source, which is a standard exponential function:

4 $$ f(t)=A{e}^{- Bt}+C $$

where *t* is time, and *A*, *B* and *C* were randomly generated from a Gaussian distribution for each observation. Then, each noisy observation was first corrected with exponential curve fitting before being introduced to a BSS filtering method.

On AP signals, which are transitory, single exponential curve fitting was applied on noisy data from time periods equivalent to AP silence intervals (see Fig. 9), which guarantees that the AP signal will not be considered by the exponential fitting routine. We defined this approach DBPC because we are providing curve fitting input from both ends of data. We also explored estimating photobleaching compensation providing data just before signal onset, which we called SBPC. Because step signals are perdurable, we can only apply SBPC on them. However, since we know step onset time, we can include the step into curve fitting as a composite function formed by exponential and step functions and provide the whole data as input. We called this approach FDPC and the fitting equation is shown below:

5 $$ f(t)=A{e}^{- Bt}+C- Eu\left(t-2.5\right) $$

where *A*, *B*, *C* and *E* are fitting parameters and *u(t-2.5)* is the step function shifted to 2.5 s.

Finally, to compare each photobleaching correction method success, we have calculated exponential parameters (*A*, *B* and *C*) relative errors. Then, to evaluate their impact on the performance of each BSS filtering method, we have calculated E_r_Amp_ and RMSE for each combination.

The codes for simulations and real data application were developed in Python 3.6.4, using Numpy 1.14, SciPy 1.0, Matplotlib 2.1.2, scikit-image 0.13.1 and scikit-learn 0.19.1 libraries. Curve fitting was implemented with SciPy ‘curve_fit’ function. PCA and ICA were implemented using scikit-learn ‘PCA’ and ‘fastICA’ functions [38]. DWT was implemented with PyWavelets 0.5.2 [39].

### Simulated imaging data

We have generated simulated imaging data based on experimental VSD imaging data and on electroporation theory to validate our method and to compare it to other established techniques.

#### Artificial video generation

The artificial video (*F*) was composed of the sum of a basal fluorescence image (*BF*), a signal (optical AP or electroporation), photobleaching and shot noise, as illustrated in the equation below:

6 $$ F\left(x,y,t\right)= BF\left(x,y\right)+ Signal\left(x,y, BF,g,t\right)+ Photobleaching\left( BF,t\right)+ Noise\left(x,y,t\right) $$

where *x* and *y* are pixel coordinates, and *g* is a gain applied to the signal to reach a desired ROI SNR. Basal fluorescence image was generated by estimating non-homogeneous illumination from experimental video by means of applying a Gaussian filter with a large kernel size (equal to the smallest spatial experimental video dimension) onto the first frame of the experimental video. Then this illumination image was normalized and multiplied by the video fluorescence grand mean.

The pure AP signal was the same as the one in the signal processing simulations. It should be modulated by *BF*, thus, it was normalized and then multiplied by *BF* in order to achieve a pure AP signal video. To build the pure electroporation signal, we collected some parameters from the experiments like cell major and minor axis radii, and intensity of the applied electric field. In addition, the same shape of cell contour in Fig. 8 was used. These parameters were used to calculate membrane potential variation after electrical field pulse application following Klee and Plonsey (1976) equations for a prolate spheroid [8, 40]. This resulted in a matrix (peak matrix) containing the expected membrane potential variation before electroporation occurred whose amplitude varied spatially depending on the angle relative to cell center and to the distance from this center. We then calculated membrane potential decay towards zero by an exponential function whose time constant was consistent with reports from the literature for similar experiments (transmembrane potential should fall to 0 mV within the first 50 ms following the shock [29]). We applied this exponential after the peak matrix and extended the number of frames to achieve a 5 s video duration consistent with experimental frame rate. Then, video was shifted by 2.5 s, added by a constant value (V_rest_ = − 80 mV) in frames before 2.5 s, normalized and multiplied by *BF*. This resulted in a pure electroporation video modulated by *BF*.

Photobleaching signal was also estimated from experimental data lacking stimuli. We tested curve fitting with several decaying functions: linear function, single exponential, a combination of an exponential and a linear function and double exponential. This last one had its initial values carefully chosen to avoid lack of convergence from the fitting algorithm. The lowest residues were obtained by the double exponential fit, consistent with the literature [41], followed closely by the combination of exponential and line. We used the double exponential function to simulate photobleaching in the artificial video.

Shot noise was based on each pixel variance through time from experimental data lacking stimuli. This variance was used as the λ value in a Poisson distribution with its mean subtracted for a final zero mean pure noise video. Each pixel had its noise generated independently.

Finally, to achieve a desired ROI SNR, pure noise video and pure signal video were segmented by MESS and each ROI signal and noise power was calculated. Based on that, a gain matrix was calculated and applied to each pure signal image pixel to modulate its amplitude towards the desired ROI SNR.

We generated 8 artificial videos this way varying the combination of following parameters: type of the signal (AP or electroporation), SNR level (either same as corresponding experimental data or -10 dB) and photobleaching presence.

#### Artificial video analysis

Artificial videos were analayzed by several fluorescence imaging established techniques and by METROID. BkS was performed by generating an extra video without signal, but containing a small amount of photobleaching drift (0.3% increase in global mean, same mean variation from experimental data). Both video with signal and video without signal were normalized by their first frames. Then, we subtracted video without signal from video with signal and reversed normalization. BkSD was performed by applying BkS followed by a standard detrending routine.

To simulate the situation of averaging videos, we generated 10 and 400 extra videos with the same signal and with the slow photobleaching drift between each of them. Then, we have averaged them and compensated photobleaching by curve fitting based on the following equations:

7 $$ f(t)=A{e}^{- Bt}+C- Dt $$

8 $$ f(t)=A{e}^{- Bt}+C- Dt- Eu\left(t-2.5\right) $$

where *A*, *B*, *C*, *D* and *E* are parameters to be estimated by non-linear curve fit.

AP video photobleaching was compensated with DBPC following (7) and electroporation photobleaching was compensated with FDPC following (8). These equations have an extra linear component if compared to those from signal processing simulations. That is to account for the fact that here photobleaching was modeled with double exponentials while there a single exponential was used. We did not use double exponentials to fix photobleaching due to frequent lack of convergence from curve fitting algorithm.

METROID uses these same equations to fix photobleaching. We applied METROID on artificial videos with three parameter configurations: default parameters (no information supplied), optimized with one selected BSS method (ICA for AP videos and PCA for electroporation videos; onset and end times supplied; manual component selection with *m* = 2 for AP videos and *m* = 3 for electroporation videos), and this same last configuration, but with the corresponding wavelet method (wICA for AP videos and wPCA for electroporation videos).

We measured E_r_Amp_, *r* and RMSE for every ROI in each artificial AP video. In artificial electroporation videos, we measured peak E_r_Amp_ (relative amplitude error at peak instant), stationary E_r_Amp_ (relative amplitude error of stationary level) and RMSE. One-way analysis of variance test with Tukey’s post-test was used when groups distribution was approximately normal, otherwise non-parametric Kruskal-Walis test with Dunn’s post-test was used. Statistical analysis was performed with GraphPad Prism 6.

### Experimental VSD imaging real data

#### Experimental protocol

Isolated adult rat cardiomyocytes were obtained by ventricles enzymatic digestion as previously described [42]. Cells were plated into a perfusion chamber with platinum electrodes in each side for cell stimulation. An epifluorescence microscope developed in our lab [43] was used to generate cell fluorescence images. An automated optical shutter was added to the microscope optical train after the light source. It was synchronized with the camera and with an electric stimulator (biphasic pulses; 0-15 V; 0.1-10 Hz; 0.1-10 ms each phase; developed at CEB/UNICAMP) in order to allow determination of electric stimulation event in the fluorescence video. Myocytes were perfused for approximately 20 min with Tyrode solution and they were paced with square bipolar electric pulses at 0.5 Hz and 5 ms each phase. A cell was chosen based on the following criteria: presence of clear striations, responding to electric stimulation by performing contractions, closely aligned with electric field and without neighboring cells or dead cell residues. A bright-field image was taken to record this situation. Perfusion was stopped and electric field threshold (E_T_) was determined by decreasing stimulus amplitude until cell stopped performing contractions. Then, VSD RH-237 (Molecular Probes, ThermoFisher Scientific, Waltham, MA) was added to extracellular solution for a final concentration of 3 μM. After that, in order to avoid motion artifacts, a contraction blocker drug, blebbistatin (Cayman Chemical, Ann Arbor, MI), was added for a final concentration of 10 μM and 20 min were waited for the drug to take effect. After that, electric stimulation was resumed to visually verify that contractions were blocked.

A few videos (2 or 3) of approximately 2.5 s (except for the last one, which lasted around 4.5 s) were recorded with a 3 min interval between each of them for each cell. The first video was recorded in absence of electric stimulation as a reference lacking cellular signal. Then, one or two subsequent videos (AP videos) were recorded with a supra-threshold bipolar electric pulse (~ 1.2xE_T_) applied 0.5 s after video beginning. In the last video (electroporation video), a stronger square monopolar electric pulse (~15xE_T_; 5 ms) from another electric stimulator (monophasic pulses; 0-150 V; 0.1-10 Hz; 0.1-10 ms; developed at CEB/UNICAMP) was applied 2.5 s after video beginning. This amplitude has been shown to be capable of eliciting [Ca^2+^] increase and oscillations consistent with increased membrane permeability caused by pore formation [29, 43] with low probability of lethality [44]. Camera was configured for video acquisition with 2 × 2 binning, 1.4 ms exposure time, 300x EM gain and windowed cell image. This configuration yielded a frame rate of 50–100 frames per second (fps) depending on window size.

#### Experimental data analysis

Cell mask was automatically generated by MESS, but sometimes manual refining was necessary to better match the bright-field image. Then, MESS divided the mask into 32 ROIs. Then METROID fixed photobleaching by either DBPC with (7) or FDPC with (8), depending on the ‘transitory’ variable.

For AP videos, METROID parameters were set as: ‘transitory’ = *True*, time of signal onset = 0.5 s, time of signal ending = 1 s, *m* = 1, *n* = 32 (as a result of configuring 32 ROIs), BSS method = ICA (later wICA with *dmey* wavelet was also tested) and automatic source selection, which makes METROID choose the component with the highest energy inside signal active interval. For electroporation videos, parameters were: ‘transitory’ = *False*, time of signal onset = 2.5 s, time of signal ending = *None*, *m* = 3, *n* = 32, BSS method = PCA (later wPCA with *Haar* wavelet was also tested) and manual source selection. SNR was estimated by computing each ROI filtered signal power divided by noise power acquired from silence intervals after photobleaching correction, but before filtering.

Filtered ROI signals are fluorescence averages in arbitrary units representing a proportion of photon counting. We have used AP amplitude as a reference to calibrate fluorescence signals to membrane potential. AP amplitude (*AP*_*amp*_) and V_rest_ values were set as 120 mV and -80 mV, respectively, taken as rounded values from the literature of patch-clamp experiments on adult isolated rat ventricular myocytes [45]. Thus membrane potential (*V(j,t)*) in each ROI *j* in electroporation experiments was then calibrated according to the following equation:

9 $$ \mathrm{V}\left(j,t\right)=\left(-\left( EP\left(j,t\right)/{AP}_{peak}(j)\right)\ast {AP}_{amp}\right)+{V}_{rest} $$

where *t* is time, *EP(j,t)* is electroporation signal in A.U. and *AP*_*peak*_*(j)* is the modulus of maximal amplitude of AP signal in A.U. in each ROI *j*. This led to a calibrated membrane potential signal in each ROI, defined by electroporation signal variation normalized by AP signal variation.

## Supplementary information

**Additional file 1. **Additional figure showing root mean square error (RMSE) results for varying SNR (from -15 dB to 5 dB in steps of 2.5 dB). (a) RMSE means as function of SNR for AP signal. Gray stars represent RMSE means calculated directly from noisy data (*N* = 120 samples for each mean), blue circles represent ICA means, red squares represent PCA means, green inverted triangles represent wICA means and orange triangles represent wPCA means. (b) RMSE means as function of SNR for step function, same color scheme as in (a). Shaded regions correspond to standard errors of the mean.

**Additional file 2.** Additional figure showing performance results for varying number of components (from 1 to 10 in unitary steps and from 10 to 30 in steps of 5). (a) Relative error for maximal AP amplitude (E_r_Amp_) means as function of number of components for AP signal. Gray stars represent E_r_Amp_ means calculated directly from noisy data (N = 120 samples for each mean), blue circles represent ICA means, red squares represent PCA means, green inverted triangles represent wICA means and orange triangles represent wPCA means. (b) Root mean square error (RMSE). (c) Pearson correlation coefficient (r) means for AP signal calculated in AP active interval. (d) E_r_Amp_ for step signal. (e) RMSE for step function. Shaded regions correspond to standard errors of the mean.

**Additional file 3.** Additional figure showing performance results for varying number of observations (from 2 to 10 in steps of 2 and from 10 to 60 in steps of 10). All graphics follow the same scheme as in Additional file 2.

**Additional file 4.** Additional figure showing photobleaching correction by different methods. (a) Gray continuous line is a noisy AP observation contaminated by noise and an exponential function representing photobleaching. Black continuous line is the exponential function, red dots are data input provided for Single Bind Photobleaching Correction (SBPC), red dot-dashed line is SBPC output, red plus blue dots are data input provided for Double Bind Photobleaching Correction (DBPC) and light blue dashed line is DBPC output. (b) Gray continuous line is a noisy step observation, black continuous line is exponential function, dark red dots are SBPC input, red dot-dashed line is SBPC output, and green dashed line is Full Data Photobleaching Correction (FDPC) output minus the step.

**Additional file 5.** Figure showing relative errors of exponential function (A*exp.(−B*t) + C) parameters for each photobleaching correction method (A as white bars, B as gray bars, and C as black bars). Lines above or below bars are standard errors of the mean. # indicates one-sample t-test statistical difference from hypothetical value zero.

**Additional file 6.** Figure showing photobleaching correction by different methods and their impacts on subsequent BSS methods performance. (a) Relative error for maximal AP amplitude (E_r_Amp_) for each BSS method after each photobleaching correction (SNR = − 10 dB). Black bars are means without exponential, grid red bars are means with exponential corrected by SBPC, and light blue vertically striped bars are means with exponential corrected by DBPC. (b) Root mean square error (RMSE) for AP after each photobleaching correction. (c) E_r_Amp_ for step for each BSS method after each photobleaching correction. Black bars and red grid bars are the same as in (a), and green horizontally striped bars are outputs from Full Data Photobleaching Correction (FDPC). FDPC Output bar is the mean relative error obtained directly from curve fit. (d) RMSE for step for each BSS method after each photobleaching compensation method. * indicates Tukey’s post-test statistical difference from that photobleaching method to all others within the same BSS method, after two-way analysis of variance statistical difference.

**Additional file 7.** Additional figure showing METROID comparison to other methods on simulated imaging data (SNR similar to corresponding experimental data, with photobleaching). (a) RMSE means for AP video (*N* = 32). (b) RMSE means for electroporation video. Bar represent means and vertical lines represent standard error of the means.

**Additional file 8.** Figure showing METROID comparison to other methods on simulated imaging data (SNR similar to corresponding experimental data, without photobleaching). (a) Representative traces in a ROI of AP filtered signal by different methods. Black circles represent true AP signal, dark gray dotted lines are ROI mean over time, gray continuous lines are BkS, light gray dashed lines are BkSD, pink dot-dashed lines are the average of 10 videos (avg10), purple dot-dashed lines are the average of 400 videos (avg400), olive green thick dashed lines are METROID output with default parameters, blue continuous lines are METROID optimized with ICA (onset and end times supplied), and green continuous lines are METROID with wICA (onset and end times supplied). (b) E_r_Amp_ means of each method (N = 32). Vertical lines represent standard error of the means. (c) *r* means. (d) RMSE means for AP video. (e) Representative traces in a ROI closer to the cathode side of electroporation filtered signal by different methods. Same color scheme as in (a), except that red continuous lines are METROID optimized with PCA (onset and end times supplied, *m* = 3), and orange continuous lines are METROID optimized with wPCA (onset and end times supplied, *m* = 3). (f) Peak E_r_Amp_ means. (g) Stationary E_r_Amp_ means. (h) RMSE means for electroporation video.

**Additional file 9.** Figure showing METROID comparison to other methods on simulated imaging data (SNR = − 10 dB in all ROIs, with photobleaching). All graphics follow the same scheme as in Additional file 8.

**Additional file 10.** Figure showing METROID comparison to other methods on simulated imaging data (SNR = − 10 dB in all ROIs, without photobleaching). All graphics follow the same scheme as in Additional file 8.

**Additional file 11.** Movie showing each ROI signal extraction in sequence, similar to Fig. 6.

## Data Availability

METROID is freely available at https://github.com/zoccoler/metroid as jupyter notebooks and a graphical interface Windows version can be downloaded at 10.6084/m9.figshare.11344046.v1.

## Abbreviations

MESS: Morphological Equal-area Standardized Segmentation
DBPC: Double Bind Photobleaching Correction
SBPC: Single Bind Photobleaching Correction
FDPC: Full Data Photobleaching Correction
