# Supplementary figures and images for: METROID: an automated method for robust quantification of subcellular fluorescence events at low SNR
Source: BMC Bioinformatics. 2020 Jul 24;21:332. doi: 10.1186/s12859-020-03661-9 (PMC7379836; doi:10.1186/s12859-020-03661-9)

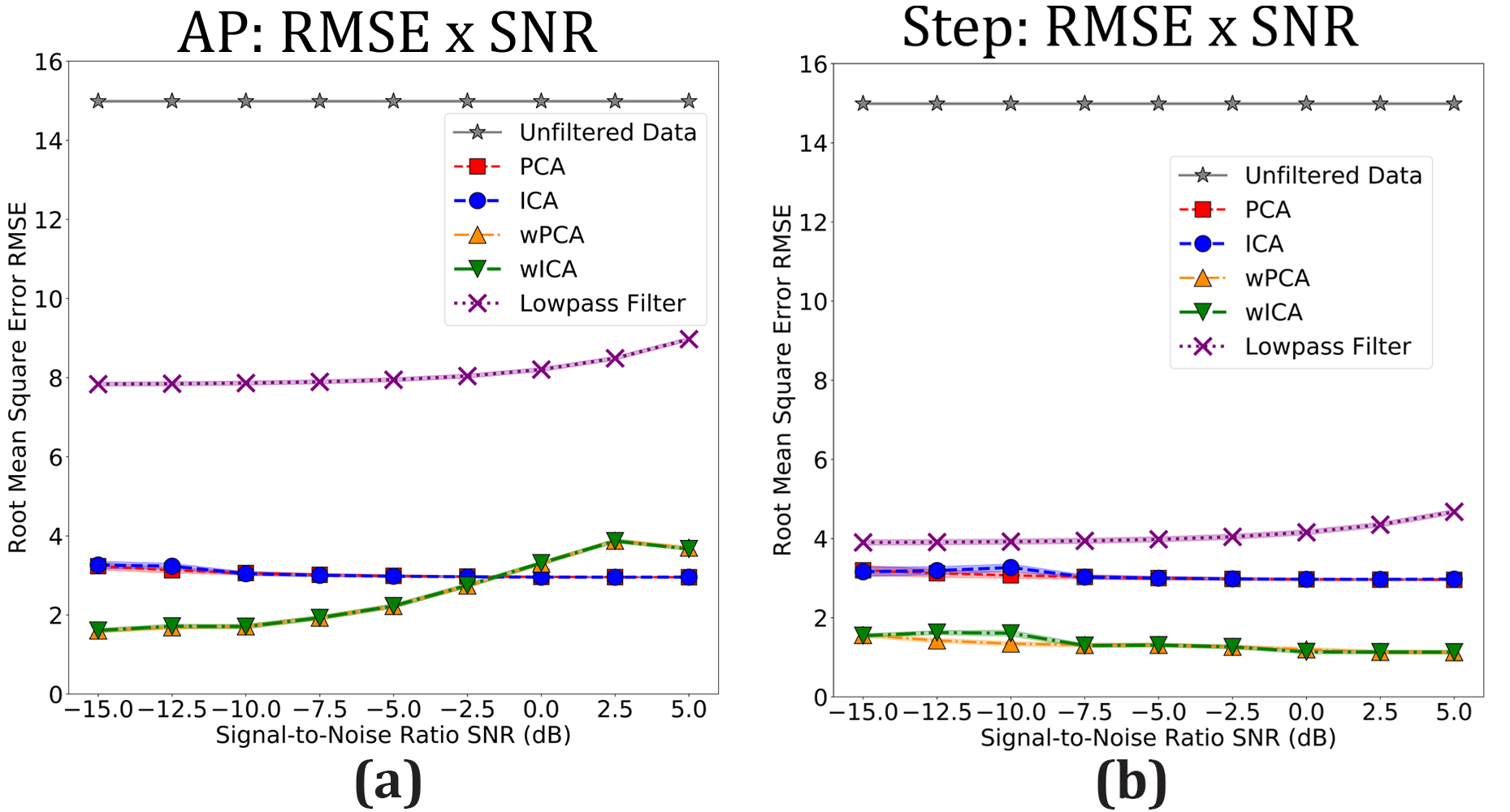

Supplement: Supplementary file 1 — Additional file 1. Additional figure showing root mean square error (RMSE) results for varying SNR (from -15 dB to 5 dB in steps of 2.5 dB). (a) RMSE means as function of SNR for AP signal. Gray stars represent RMSE means calculated directly from noisy data (N = 120 samples for each mean), blue circles represent ICA means, red squares represent PCA means, green inverted triangles represent wICA means and orange triangles represent wPCA means. (b) RMSE means as function of SNR for step function, same color scheme as in (a). Shaded regions correspond to standard errors of the mean. [file 12859_2020_3661_MOESM1_ESM.tif]

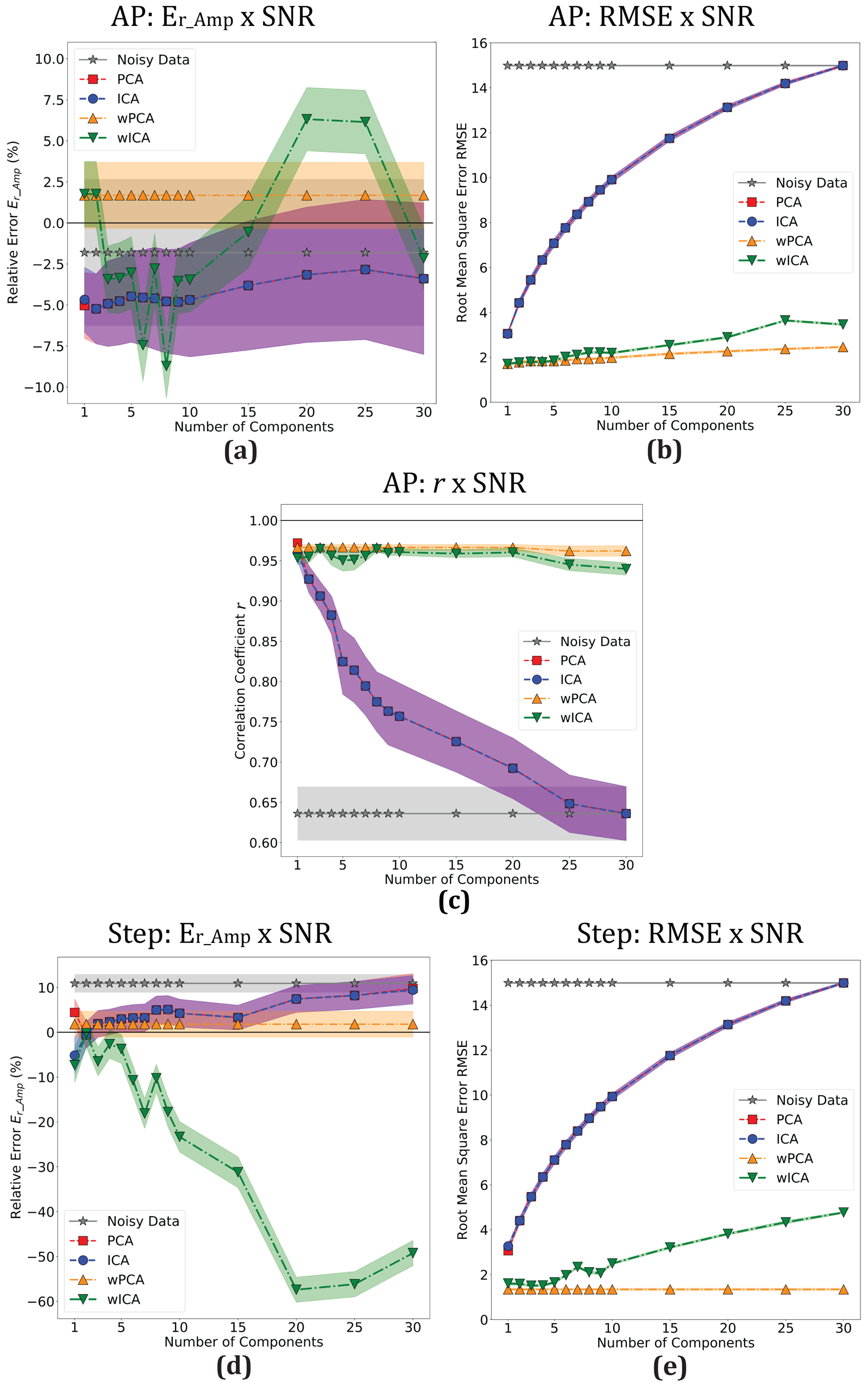

Supplement: Supplementary file 2 — Additional file 2. Additional figure showing performance results for varying number of components (from 1 to 10 in unitary steps and from 10 to 30 in steps of 5). (a) Relative error for maximal AP amplitude (Er_Amp) means as function of number of components for AP signal. Gray stars represent Er_Amp means calculated directly from noisy data (N = 120 samples for each mean), blue circles represent ICA means, red squares represent PCA means, green inverted triangles represent wICA means and orange triangles represent wPCA means. (b) Root mean square error (RMSE). (c) Pearson correlation coefficient (r) means for AP signal calculated in AP active interval. (d) Er_Amp for step signal. (e) RMSE for step function. Shaded regions correspond to standard errors of the mean. [file 12859_2020_3661_MOESM2_ESM.tif]

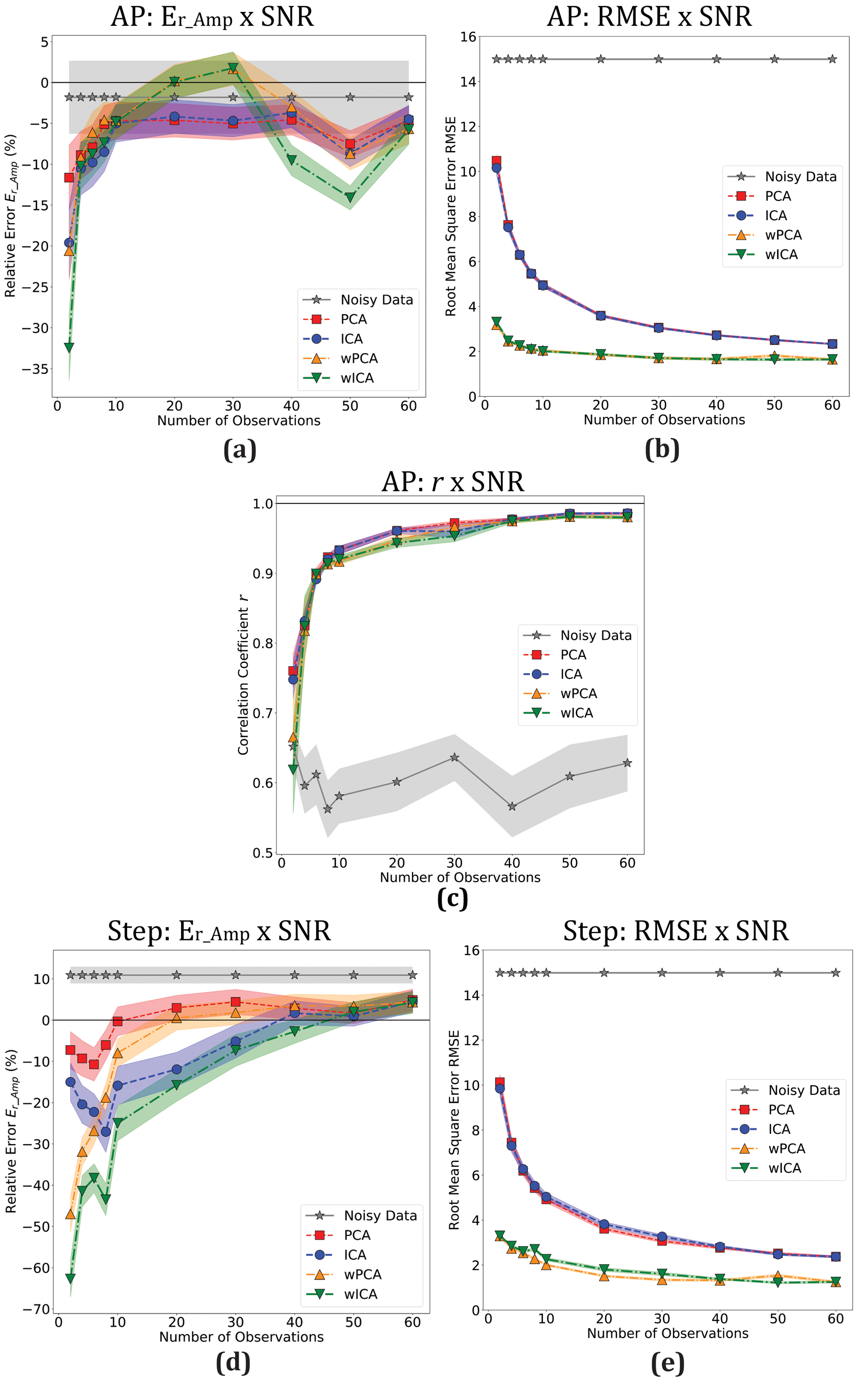

Supplement: Supplementary file 3 — Additional file 3. Additional figure showing performance results for varying number of observations (from 2 to 10 in steps of 2 and from 10 to 60 in steps of 10). All graphics follow the same scheme as in Additional file 2. [file 12859_2020_3661_MOESM3_ESM.tif]

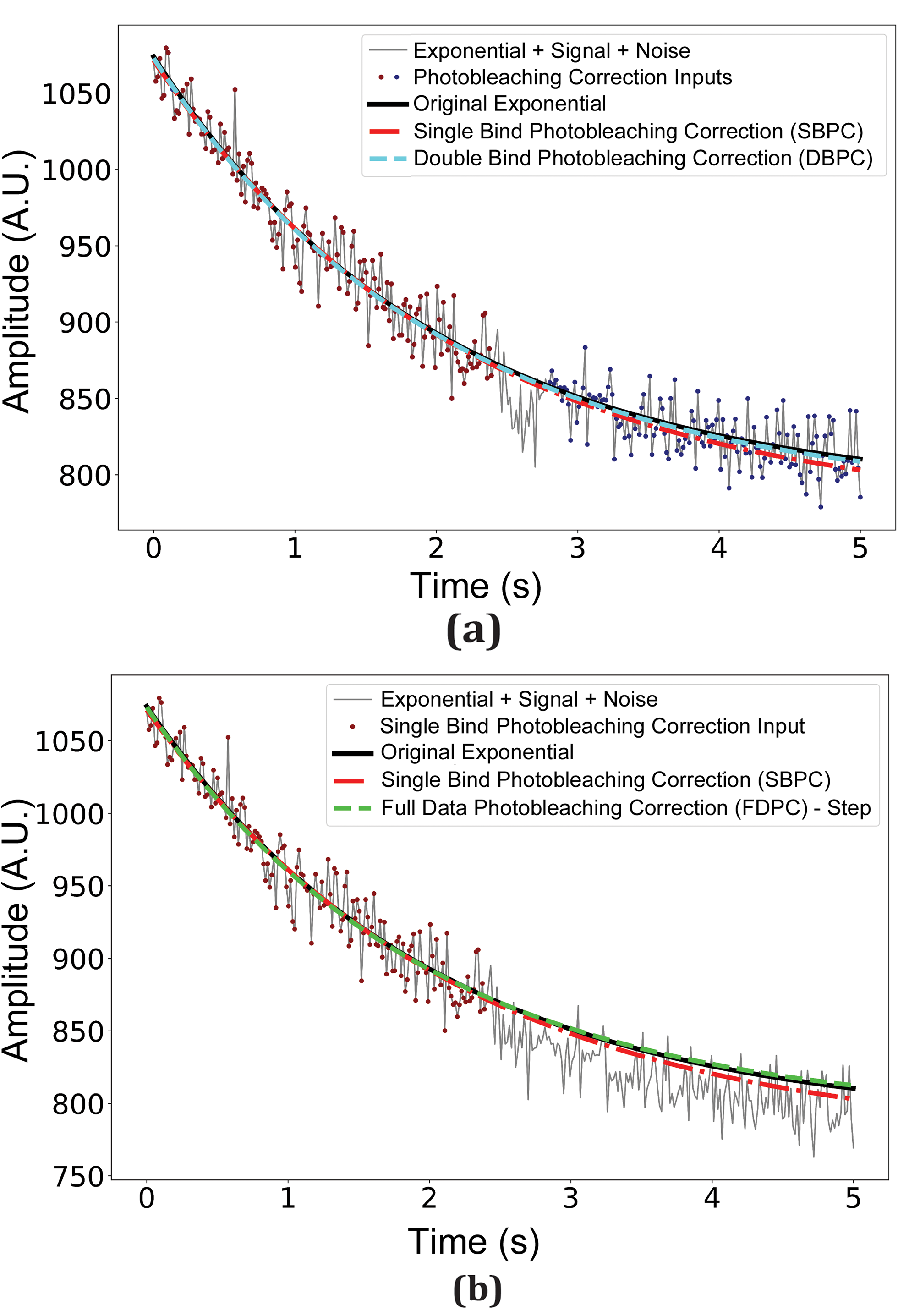

Supplement: Supplementary file 4 — Additional file 4. Additional figure showing photobleaching correction by different methods. (a) Gray continuous line is a noisy AP observation contaminated by noise and an exponential function representing photobleaching. Black continuous line is the exponential function, red dots are data input provided for Single Bind Photobleaching Correction (SBPC), red dot-dashed line is SBPC output, red plus blue dots are data input provided for Double Bind Photobleaching Correction (DBPC) and light blue dashed line is DBPC output. (b) Gray continuous line is a noisy step observation, black continuous line is exponential function, dark red dots are SBPC input, red dot-dashed line is SBPC output, and green dashed line is Full Data Photobleaching Correction (FDPC) output minus the step. [file 12859_2020_3661_MOESM4_ESM.tif]

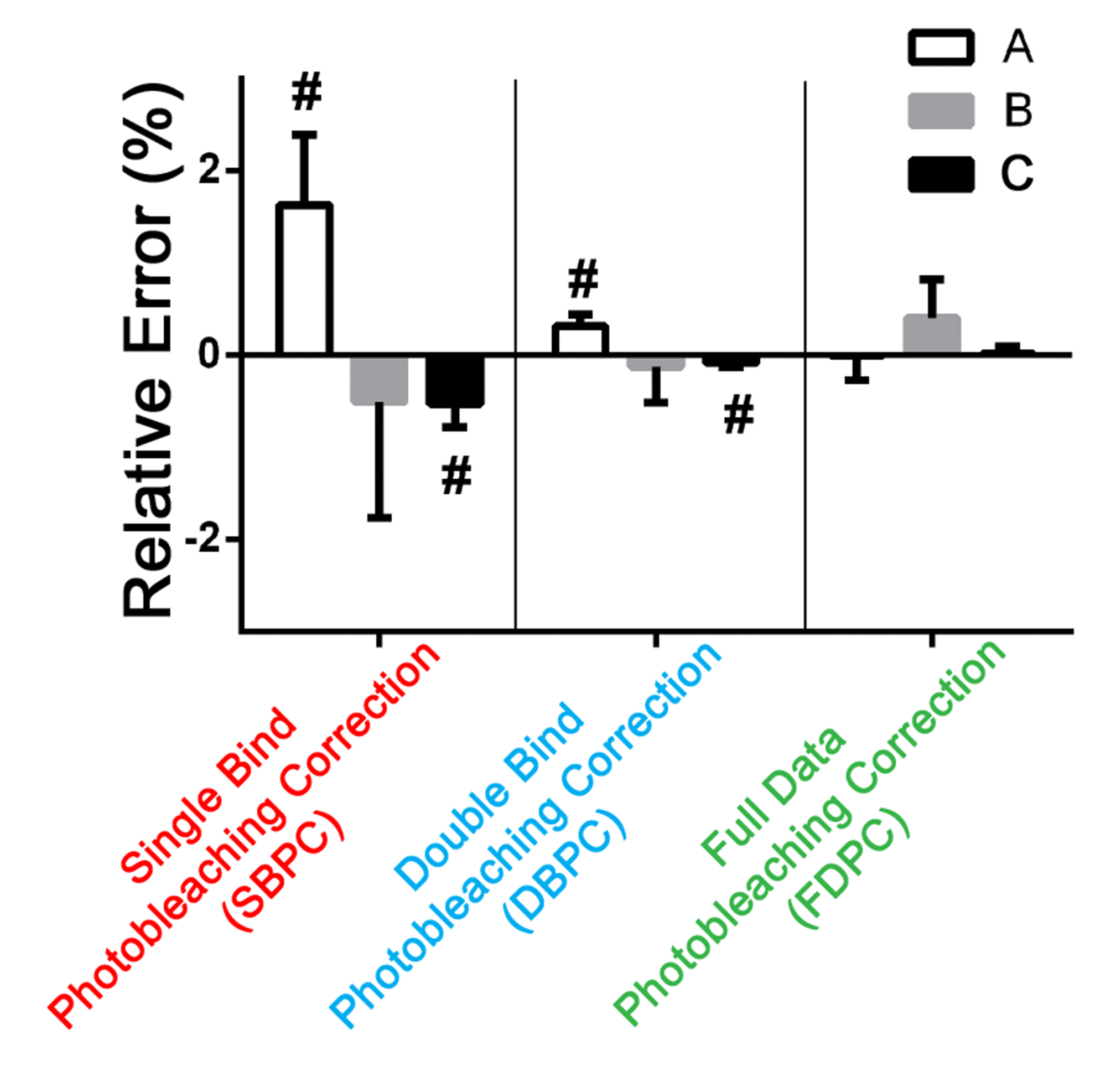

Supplement: Supplementary file 5 — Additional file 5. Figure showing relative errors of exponential function (A*exp.(−B*t) + C) parameters for each photobleaching correction method (A as white bars, B as gray bars, and C as black bars). Lines above or below bars are standard errors of the mean. # indicates one-sample t-test statistical difference from hypothetical value zero. [file 12859_2020_3661_MOESM5_ESM.tif]

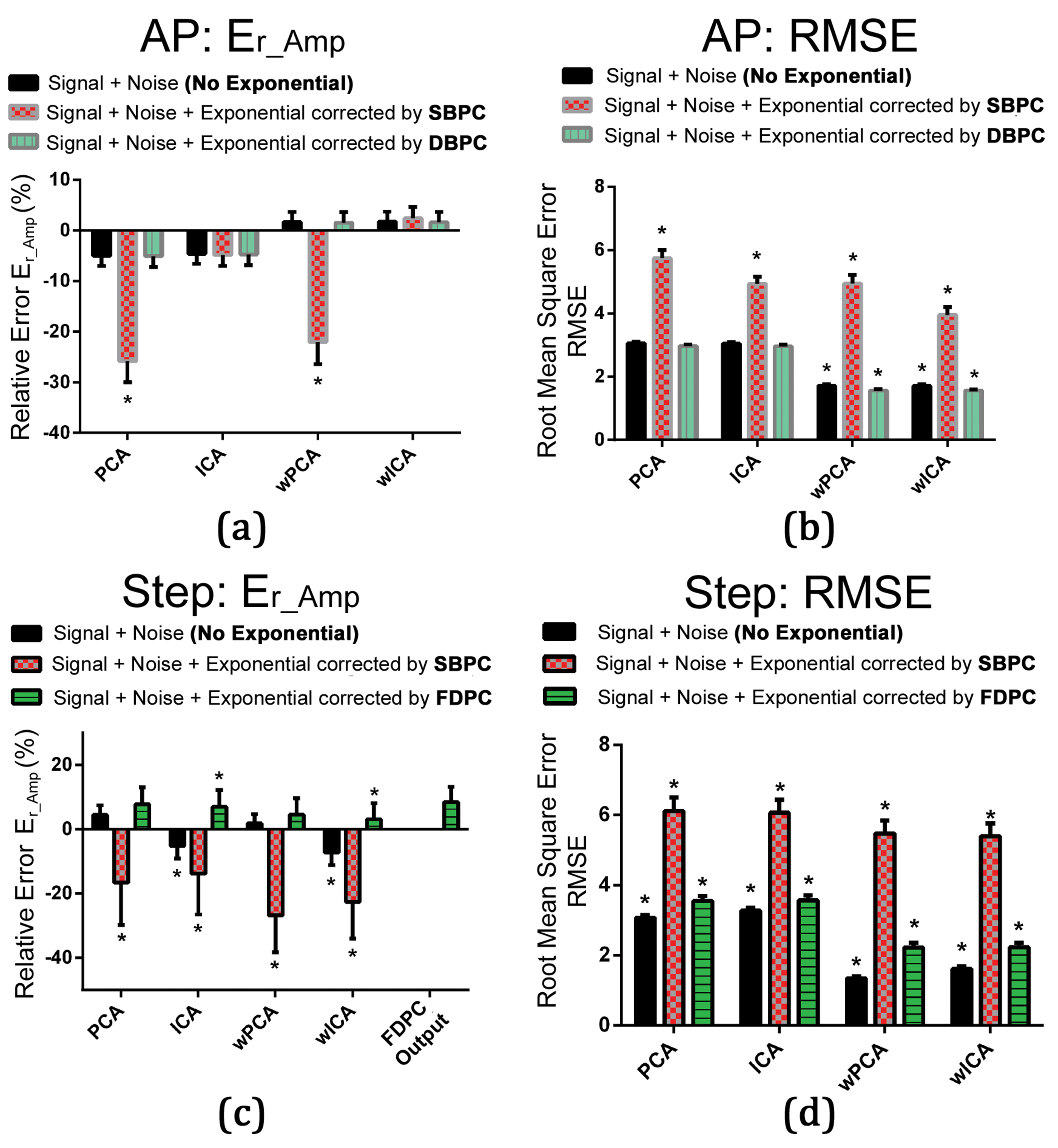

Supplement: Supplementary file 6 — Additional file 6. Figure showing photobleaching correction by different methods and their impacts on subsequent BSS methods performance. (a) Relative error for maximal AP amplitude (Er_Amp) for each BSS method after each photobleaching correction (SNR = − 10 dB). Black bars are means without exponential, grid red bars are means with exponential corrected by SBPC, and light blue vertically striped bars are means with exponential corrected by DBPC. (b) Root mean square error (RMSE) for AP after each photobleaching correction. (c) Er_Amp for step for each BSS method after each photobleaching correction. Black bars and red grid bars are the same as in (a), and green horizontally striped bars are outputs from Full Data Photobleaching Correction (FDPC). FDPC Output bar is the mean relative error obtained directly from curve fit. (d) RMSE for step for each BSS method after each photobleaching compensation method. * indicates Tukey’s post-test statistical difference from that photobleaching method to all others within the same BSS method, after two-way analysis of variance statistical difference. [file 12859_2020_3661_MOESM6_ESM.tif]

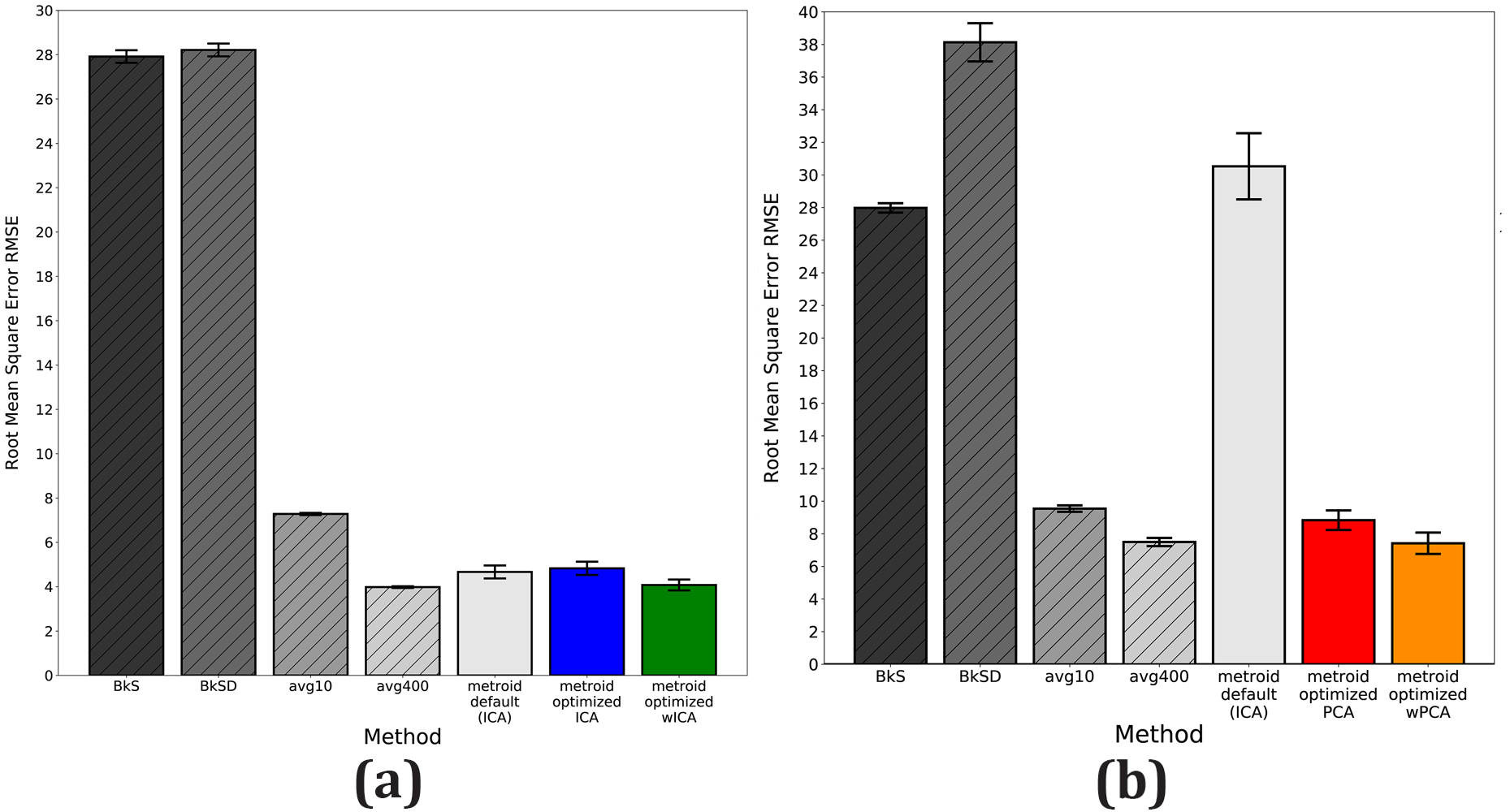

Supplement: Supplementary file 7 — Additional file 7. Additional figure showing METROID comparison to other methods on simulated imaging data (SNR similar to corresponding experimental data, with photobleaching). (a) RMSE means for AP video (N = 32). (b) RMSE means for electroporation video. Bar represent means and vertical lines represent standard error of the means. [file 12859_2020_3661_MOESM7_ESM.tif]

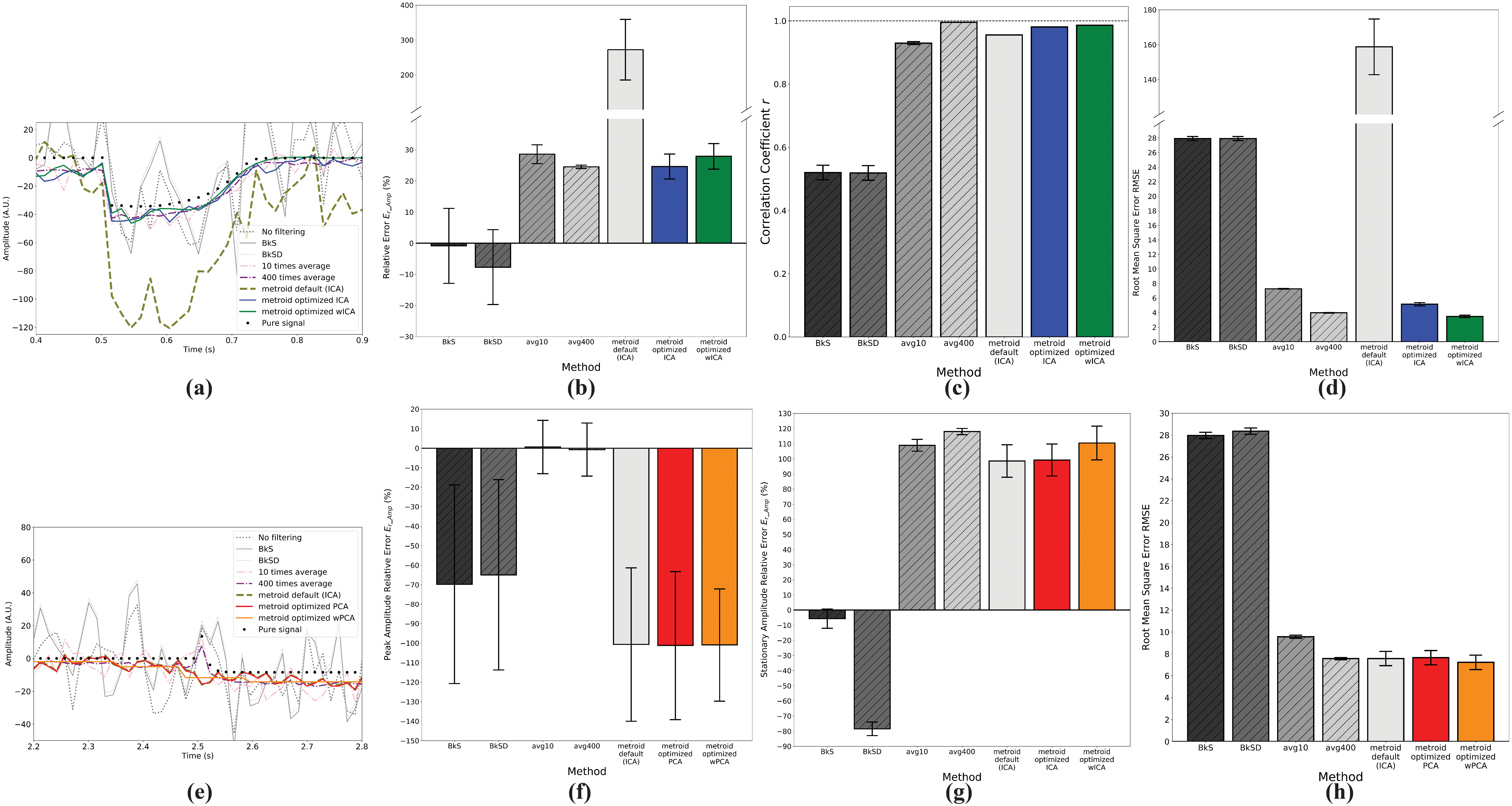

Supplement: Supplementary file 9 — Additional file 9. Figure showing METROID comparison to other methods on simulated imaging data (SNR = − 10 dB in all ROIs, with photobleaching). All graphics follow the same scheme as in Additional file 8. [file 12859_2020_3661_MOESM9_ESM.tif]
